# New species, additions and a key to the Brazilian species of the Geminata clade of *Solanum* L. (Solanaceae) in Brazil

**DOI:** 10.3897/phytokeys.47.9076

**Published:** 2015-03-10

**Authors:** Sandra Knapp, João Renato Stehmann, Leandro L. Giacomin

**Affiliations:** 1 Department of Life Sciences, Natural History Museum, Cromwell Road, London SW7 5BD, United Kingdom; 2 Instituto de Ciências Biológicas, Departamento de Botânica, Laboratório de Sistemática Vegetal, Universidade Federal de Minas Gerais – UFMG, Av. Antônio Carlos, 6627, Pampulha, Belo Horizonte, CEP 31270-901, MG, Brazil; 3 (current address) Instituto de Ciências e Tecnologia das Águas and Herbário HSTM, Universidade Federal do Oeste do Pará - UFOPA, Av. Mendonça Furtado, 2946, Santarém, CEP 68040-050, PA, Brazil

**Keywords:** Atlantic forests, diversity, endemism, assessment of extinction risk

## Abstract

Two additions and four new species are described from Brazil for the large Geminata clade (*Solanum*: Solanaceae) bringing the total diversity in the group to 149 species, with 44 of these occurring in Brazil. New species are described from Brazil: *Solanum amorimii* S.Knapp & Giacomin, **sp. nov.** from Bahia and adjacent Minas Gerais states, *Solanum filirhachis* Giacomin & Stehmann, **sp. nov.** from Espirito Santo, *Solanum psilophyllum* Stehmann & Giacomin, **sp. nov.** from Minas Gerais and *Solanum verticillatum* S.Knapp & Stehmann, **sp. nov.** from São Paulo, Rio de Janeiro and Minas Gerais. Modern character-rich descriptions and lectotypifications are provided for *Solanum apiahyense* Witasek and *Solanum lacteum* Vell. All are illustrated, mapped and assessed for conservation status. We also provide a brief analysis of the diversity and endemism of the Geminata clade in Brazil and a key to all 44 Brazilian species.

## Abstract (pt)

Duas novas adições e quatro novas espécies ocorrentes no Brasil são apresentadas para o clado Geminata (*Solanum*: Solanaceae), aumentando a diversidade conhecida para grupo para 149 espécies, das quais 44 ocorrem no Brasil. Táxons novos são descritos: *Solanum amorimii* S.Knapp & Giacomin, **sp. nov.**, conhecido para os estados da Bahia e Espírito Santo, *Solanum filirhachis* Giacomin & Stehmann, **sp. nov.** conhecido para o Espirito Santo, *Solanum psilophyllum* Stehmann & Giacomin, **sp. nov.** conhecido para Minas Gerais e *Solanum verticillatum* S.Knapp & Stehmann, **sp. nov.** conhecido para São Paulo, Rio de Janeiro and Minas Gerais. Descrições detalhadas e lectotipificações são apresentadas para *Solanum lacteum* Vell. e *Solanum apiahyense* Witasek. Para todos os táxons são apresentados imagens, mapas e categorias de ameaça. É também apresentada uma breve análise de diversidade e endemismo do clado Geminata no Brasil, além de uma chave dicotômica para as 44 espécies ocorrentes no país.

## Introduction

*Solanum* L. is one of the largest of flowering plant genera, and includes *ca.* 1400 species occurring worldwide on all continents except Antarctica. The genus was traditionally divided into the “spiny” and “non-spiny” solanums (e.g., Dunal 1852), based on the presence or absence of leaf and stem prickles. Molecular phylogenetic analysis showed that the “spiny” solanums form a monophyletic group (Bohs 2005; Weese and Bohs 2007; Särkinen et al. 2013), but the “non-spiny” solanums consist of a grade comprising several distinct monophyletic groups. The largest of these monophyletic groups are the potato clade (*ca.* 178 species of potatoes and their relatives), the “M” clade (of Särkinen et al. 2013; *ca.* 110 species of true nightshades and dulcamaroids; see Knapp 2013) and the Geminata clade, whose Brazilian members are treated here.

The Geminata clade as broadly defined contains 149 species, all but one of which occur in the New World tropics (Knapp 2002a, 2008). Members of the group are shrubs and small trees mostly occurring in forest understory habitats; they are often inconspicuous, rare and rarely collected, with only a few widespread and weedy species. The group’s name comes from the morphology of sympodial units in many of the component species where leaves appear to be twinned (geminate) at a node due to concaulescence of shoot generations (Danert 1958). The two leaves are often of markedly different sizes and occasionally even shapes (see Knapp 2002a); plants are then markedly anisophyllous. Knapp (2002a) treated the group as section *Geminata* (G.Don) Walp. and divided the group into several informal species groups based on seed and sympodial morphology. Species later found to belong to the monophyletic group containing members of section *Geminata* (e.g., *Solanum argentinum* Bitter & Lillo and *Solanum havanense* Jacq. and its relatives; Weese and Bohs 2007) were added to the group and a list of component species with a key to all taxa was provided (Knapp 2008).

*Solanum trachytrichium* Bitter was included in Geminata by Knapp (2002a, 2008) but recent molecular work in the related Brevantherum clade (Giacomin 2015) revealed that it, plus the rare Brazilian species *Solanum apiahyense* Witasek (Giacomin and Stehmann 2014) are sister to the Geminata clade as treated by Knapp (2008), but with low support. *Solanum apiahyense* together with *Solanum trachytrichium* form a strongly supported lineage that is either sister to the Brevantherum or Geminata clade depending on the marker used. We here include these two taxa in the broad circumscription of the Geminata clade for practical reasons of identification and morphological similarity, while recognising that future phylogenetic studies may show *Solanum apiahyense* and *Solanum trachytrichium* to be a distinct group (see discussion under *Solanum apiahyense*). They share trichome types with the Geminata clade, and their possible relationships and similarities are discussed below.

An analysis of species richness and endemism patterns in the Neotropics using a group of species including many members of the Geminata clade (Knapp 2002b) showed peaks of both diversity and endemism in the Andes and south-eastern Brazil, as had been predicted by Gentry (1982) for understory plants in general. Subsequent analysis on a country level (Knapp 2008) showed species richness of the Geminata clade was highest in Colombia, Peru and Brazil with 41 (9 endemic from Colombia, 22%; 11 endemic from Peru, 27%) and 35 (incorrectly recorded as 34; 17 endemic, 50%) species respectively. Concentrated work in Brazil focused on the *Lista de Especies de Flora do Brasil* (Stehmann et al. 2014) has clarified the status of several names of dubious application and brought to light new species of the Geminata clade that are described here. We here record 44 species (43 native) of the group for Brazil, instead of 35 recorded by Knapp (2008). Of these nine additional records for the country, two are range extensions (*Solanum arboreum* Dunal, *Solanum diphyllum* L.) and are documented with character-rich descriptions in the literature cited above, while two have not been described over their entire range (*Solanum apiahyense* and *Solanum lacteum*) or have been of uncertain application (*Solanum lacteum*) and we provide descriptions to a modern standard here. Five new species have been discovered since Knapp (2008). We describe four of these new taxa (one is in review elsewhere, see below) and clarify diversity and distribution of the entire clade for Brazil.

## Materials and methods

Descriptions are based on field observations and examination of herbarium specimens from 27 collections in Brazil and abroad (B, BM, BHCB, BR, CEPEC, CORD, ESA, F, FUEL, FURB, G, HUEFS, IAC, JPB, K, LE, MBM, MBML, NY, PMSP, RB, SP, SPSF, UEC, UT, VIC, WU). Herbarium acronyms are from Index Herbariorum (http://sciweb.nybg.org/science2/IndexHerbariorum.asp) and all specimens are cited in the text. Full data are provided in the supplemental file and on the Solanaceae Source website (http://www.solanaceaesource.org). Extent of Occurrence (EOO) and Area of Occupancy (AOO) were calculated using GeoCat (http://geocat.kew.org) using the standard 2 km^2^ cell width for AOO calculation. Conservation status of each species was assessed using the IUCN (2014) criteria based on the GeoCat analyses (Bachman et al. 2012) combined with field knowledge.

## Results and discussion

The broadly defined Geminata clade has 43 species native to Brazil (Table 1); only *Solanum diphyllum* (see Knapp 2002a) is known only from cultivation and may be naturalising. The state distribution of each species is given in Table 1, along with endemic status and extra-Brazilian distribution of non-endemic species. Endemism of native Brazilian Geminata species now stands at 65% (28/43 native species, excluding *Solanum diphyllum*). The south-eastern region (following Brazilian political divisions) is the most species-rich area of the country with 24 species, followed by the southern region (19 species); the northern regions have fewer species, reflecting the circum-Amazonian species richness of *Solanum* in general (Table 2). The distribution in federal units (states) by species is presented in the second column of Table 1 and by state in Table 3. The states of Paraná (19 species), Minas Gerais (17 species) and Santa Catarina (16 species) are the most species-rich, followed by São Paulo (15 species) and Rio de Janeiro (12 species). Six species are endemic to a single state; *Solanum cordioides* and *Solanum santosii* in Bahia, *Solanum filirhachis* in Espirito Santo, *Solanum psilophyllum* in Minas Gerais, *Solanum gertii* in Paraná, and *Solanum* sp. *1* (a new species based on collections including *Giacomin et al. 1789* [BHCB, UFP] being described by M.F. Agra and currently in review) in Pernambuco.

**Table 1.** Brazilian species of the Geminata clade (country endemics are in bold face); of 43 native species (is *Solanum diphyllum* introduced) 28 are endemic to Brazil. Brazilian states are abbreviated following *Lista de Especies de Flora do Brasil* (Stehmann et al. 2014) – see also Table 3.

Species	Brazilian distribution	Extra-Brazilian distribution	Biome distribution in Brazil
***Solanum alatirameum* Bitter**	PR; RS; SC		Mata Atlântica
***Solanum amorimii* S.Knapp & Giacomin**	BA; MG		Mata Atlântica
*Solanum anisophyllum* van Heurck & Mull.-Arg.	AC; AM	Ecuador, Peru	Amazônia
***Solanum apiahyense* Witasek**	PR; SC; SP		Mata Atlântica
***Solanum arenarium* Sendtn.**	RJ; RS		Mata Atlântica
*Solanum arboreum* Dunal	RR		Amazônia
***Solanum bahianum* S.Knapp**	BA; ES; MG		Mata Atlântica
***Solanum caavurana* Vell.**	AL; BA; CE; ES; MA; MG; MS; MT; PB; PE; PI; PR; RJ; RN; SC; SE; SP		Caatinga, Cerrado, Mata Atlântica
*Solanum campaniforme* Roem. & Schult.	AM; BA; CE; DF; ES; MA; MG; PA; PB; PE; PR; RJ; RR; RS; SC; SP	Venezuela	Amazônia, Caatinga, Cerrado, Mata Atlântica
***Solanum canoasense* L.B.Sm. & Downs**	PR; SC		Mata Atlântica
***Solanum cassioides* L.B.Sm. & Downs**	MG; PR; RS; SC		Mata Atlântica
*Solanum compressum* L.B.Sm. & Downs	PR; RS; SC	Argentina, Paraguay	Amazônia, Cerrado, Mata Atlântica
***Solanum cordioides* S.Knapp**	BA		Mata Atlântica
*Solanum corumbense* S.Moore	MS; MT; RO	Bolivia, Paraguay	Mata Atlântica
*Solanum delicatulum* L.B.Sm. & Downs	PR; RS; SC; SP	Argentina, Paraguay	Mata Atlântica
*Solanum diphyllum* L.	MG	Introduced from Central America; cultivated and escaped worldwide	Cultivated
***Solanum evonymoides* Sendtn.**	BA; ES; MG		Mata Atlântica
***Solanum filirhachis* Giacomin & Stehmann**	ES		Mata Atlântica
***Solanum gertii* S.Knapp**	PR		Mata Atlântica
***Solanum gnaphalocarpon* Vell.**	MG; PR; RJ; SP		Mata Atlântica
***Solanum intermedium* Sendtn.**	MG; RJ; SP		Mata Atlântica, Cerrado
***Solanum kleinii* L.B.Sm. & Downs**	PR; SC; SP		Mata Atlântica
***Solanum lacteum* Vell.**	ES; MG; RJ		Mata Atlântica
*Solanum leptopodum* van Heurck & Mull.-Arg.	AM	Ecuador, Peru	Amazônia
*Solanum leucocarpon* Dunal	AC; AM; GO; MA; MG; MT; PA; RO; RR	Panama, Colombia, Venezuela, Ecuador, Peru	Amazônia, Cerrado, Mata Atlântica
*Solanum nudum* Dunal	AC; AM	Central and South America	Amazônia
*Solanum oppositifolium* Ruiz & Pavon	AC; AM; PA; RR	Ecuador, Peru	Amazônia
***Solanum pabstii* L.B.Sm. & Downs**	PR; RS; SC; SP		Mata Atlântica
*Solanum pseudocapsicum* L.	DF; ES; GO; MG; MS; MT; PR; RJ; RS; SC; SP	Cultivated worldwide	Cerrado, Mata Atlântica
***Solanum pseudodaphnopsis* L.A.Mentz & Stehmann**	PR; SC; SP		Mata Atlântica
*Solanum pseudoquina* A.St.Hil.	BA; ES; MG; PR; RJ; RS; SC; SP	Argentina, Paraguay	Mata Atlântica
***Solanum psilophyllum* Stehmann & Giacomin**	MG		Mata Atlântica, Cerrado
***Solanum reitzii* L.B.Sm. & Downs**	PR; RS; SC		Mata Atlântica
***Solanum restingae* S.Knapp**	BA; ES; RJ		Mata Atlântica
*Solanum robustifrons* Bitter	AC; AM	Peru, Ecuador, Colombia, Bolivia	Amazônia
***Solanum santosii* S.Knapp**	BA		Mata Atlântica
*Solanum sessile* Ruiz & Pav.	AC; AM	Peru, Ecuador, Colombia, Bolivia	Amazônia
***Solanum* sp. 1**	PE	[in press Agra]	Mata Atlântica
***Solanum spissifolium* Sendtn.**	SP		Mata Atlântica
***Solanum stipulatum* Vell.**	BA; ES; MG; PR; RJ; SC; SP		Mata Atlântica
*Solanum symmetricum* Rusby	MG; MT; PR	Bolivia	Mata Atlântica
*Solanum trachytrichium* Bitter	PR; RS; SC; SP	Argentina, Paraguay	Mata Atlântica
***Solanum verticillatum* S.Knapp & Stehmann**	MG; RJ; SP		Mata Atlântica
***Solanum warmingii* Hiern**	BA; ES; MG; RJ		Mata Atlântica

**Table 2.** Species of the Geminata clade and their distribution in the regions of Brazil (as defined in List of Species of the Brazilian Flora. Rio de Janeiro Botanical Garden. http://floradobrasil.jbrj.gov.br/ [Accessed on: 08 Nov. 2014]

Region	Species
Central-West (5)	caavurana, campaniforme, corumbense, leucocarpon, pseudocapsicum
North-Eeast (12)	bahianum, caavurana, campaniforme, cordioides, evonymoides, leucocarpon, pseudoquina, restingae, sp. 1, santosii, stipulatum, warmingii
North (10)	anisophyllum, arboreum, campaniforme, corumbense, leptopodum, leucocarpon, nudum, oppositifolium, robustifrons, sessile
South-East (24)	apiahyense, arenarium, bahianum, caavurana, campaniforme, cassioides, delicatulum, evonymoides, gnaphalocarpon, intermedium, kleinii, lacteum, leucocarpon, pabstii, pseudocapsicum, pseudodaphnopsis, pseudoquina, psilophyllum, restingae, spissifolium, stipulatum, symmetricum, trachytrichium, warmingii
South (19)	alatirameum, apiahyense, arenarium, caavurana, campaniforme, canoasense, cassioides, compressum, delicatulum, gertii, gnaphalocarpon, kleinii, pabstii, pseudocapsicum, pseudodaphnopsis, pseudoquina, reitzii, stipulatum, symmetricum, trachytrichium

**Table 3.** Species of the Geminata clade occurring in each of the 27 Brazilian states (incl. DF). Species endemic to that state are in boldface type.

State	#	Species
Acre (AC)	6	anisophyllum, leucocarpon, nudum, oppositifolium, robustifrons, sessile
Alagoas (AL)	1	caavurana
Amapá (AP)	1	leucocarpon
Amazonas (AM)	8	anisophyllum, campaniforme, leptopodum, leucocarpon, nudum, oppositifolium, robustifrons, sessile
Bahia (BA)	10	amorimii, caavurana, campaniforme, **cordioides**, evonymoides, pseudoquina, restingae, **santosii**, stipulatum, warmingii
Ceará (CE)	2	caavurana, campaniforme
Distrito Federal (DF)	2	campaniforme, pseudocapsicum
Espirito Santo (ES)	9	bahianum, caavurana, campaniforme, evonymoides, **filirhachis**, lacteum, restingae, pseudoquina, warmingii
Goias (GO)	3	leucocarpon, pseudocapsicum, stipulatum
Maranhão (MA)	3	caavurana, campaniforme, pseudocapsicum
Mato Grosso (MT)	5	caavurana, corumbense, leucocarpon, pseudocapsicum, symmetricum
Mato Grosso do Sul (MS)	2	caavurana, corumbense
Minas Gerais (MG)	17	amorimii, caavurana, campaniforme, cassioides, diphyllum, evonymoides, gnaphalocarpon, intermedium, lacteum, leucocarpon, pseudocapsicum, pseudoquina, **psilophyllum**, stipulatum, symmetricum, verticillatum, warmingii
Pará (PA)	3	caavurana, campaniforme, leucocarpon
Paraíba (PB)	2	caavurana, campaniforme
Paraná (PR)	19	alatirameum, apiahyense, caavurana, campaniforme, canoasense, cassioides, compressum, delicatulum, **gertii**, gnaphalocarpon, kleinii, pabstii, pseudocapsicum, pseudodaphnopsis, pseudoquina, reitzii, stipulatum, symmetricum, trachytrichium
Pernambuco (PE)	3	caavurana, campaniforme, **sp. 1**
Piauí (PI)	1	caavurana
Rio de Janeiro (RJ)	12	arenarium, caavurana, campaniforme, gnaphalocarpon, intermedium, lacteum, pseudocapsicum, pseudoquina, restingae, stipulatum, verticillatum, warmingii
Rio Grande do Norte (RN)	1	caavurana
Rio Grando do Sul (RS)	11	alatirameum, arenarium, campaniforme, cassioides, compressum, delicatulum, pabstii, pseudocapsicum, pseudoquina, reitzii, trachytrichium
Rôndonia (RO)	2	corumbense, leucocarpon
Roraima (RR)	3	arboreum, campaniforme, leucocarpon
Santa Catarina (SC)	16	alatirameum, apiahyense, caavurana, campaniforme, canoasense, cassioides, compressum, delicatulum, kleinii, pabstii, pseudocapsicum, pseudodaphnopsis, pseudoquina, reitzii, stipulatum, trachytrichium
São Paulo (SP)	15	apiahyense, caavurana, campaniforme, delicatulum, gnaphalocarpon, intermedium, kleinii, pabstii, pseudocapsicum, pseudodaphnopsis, pseudoquina, spissifolium, stipulatum, trachytrichium, verticillatum
Sergipe (SE)	1	caavurana
Tocantins (TO)	0	---

Only seven of the native species occur exclusively outside the Mata Atlântica biome (Atlantic rainforest; as defined by IBGE 2012); all of these are Amazonian (see Table 1). All of the endemic species (28) occur in Mata Atlântica, with 24 of those occurring only in that biome; only *Solanum caavurana* (Caatinga + Cerrado), *Solanum intermedium* (Cerrado) and *Solanum psilophyllum* (Cerrado; in the forested *capões* associated with *Campos Rupestres*) occur in other vegetation types. *Solanum caavurana* is widespread in secondary habitats and *Solanum intermedium* and *Solanum psilophyllum* occur in regions where the Cerrado and Mata Atlântica meet (e.g., Serra do Cipó in Minas Gerais). Few Geminata species are widespread in Brazil; only *Solanum caavurana*, *Solanum campaniforme*, *Solanum leucocarpon*, *Solanum pseudocapsicum*, *Solanum pseudoquina* and *Solanum stipulatum* occur in more than four states.

Because of their biology and occurrence in small populations of scattered individuals, most of the species described here (with the exception of *Solanum verticillatum*) can be classified as rare and of some conservation concern. Rabinowitz (1981) suggested that species become rare (and by extension subject to extinction risk) by a variety of pathways and if this were so, the ecological and evolutionary consequences of rarity would be diverse. She analysed plant rarity using a scheme that took into account range size, habitat specificity and local abundance (population size); in her classification rare species ranged from ‘common’ to ‘endemics’. The ecological consequences of rarity are likely to differ in rare taxa of the different categories.

Although the south-eastern part of Brazil is the most intensively collected part of the country (Sousa-Baena et al. 2013) all of the new species and additions to the Geminata for the Brazilian flora come from this region. As collecting is intensified in other regions (such as the western edges of the Amazon basin) we expect more of these forest understory solanums for Brazil.

## Taxonomic treatment of new species

### Solanum amorimii

Taxon classificationPlantaeSolanalesSolanaceae

S.Knapp & Giacomin sp. nov.

urn:lsid:ipni.org:names:77145587-1

Figures 1A, B , 2

#### Diagnosis.

Like *Solanum restingae* S.Knapp but differing in smaller flowers with narrowly deltate to long-triangular calyx lobes, unwinged stems and usually somewhat auriculate leaves.

#### Type.

Brazil. Bahia: Mun. Tancredo Neves, Estrada para os distritos de Água Branca e Julião, *ca.* 14. 1 km de Tancredo Neves, 554 m, 13°26'36"S, 39°30'40"W, 12 Sep 2005 (fl), *A.M. Amorim, J. Jardim, J. Paixåo, S. Sant’Ana & E. dos Santos 5210* (holotype: CEPEC [CEPEC-110253]; isotypes: BHCB [BHCB002643, BHCB019062]).

#### Description.

Shrub to small treelet 0.5–3 m tall; young stems terete, glabrous or minutely puberulent with simple uniseriate trichomes to 0.5 mm long; new growth glabrous; bark of older stems smooth, greenish brown. Sympodial units difoliate, geminate; leaves of a pair not differing in shape. Leaves simple, the major leaves 8–10(-15) cm long, 2–3(-5) cm wide, elliptic to obovate, usually widest near the middle or in the distal half, glabrous on both surfaces, fleshy in texture; primary veins 8 pairs, usually paler than the lamina; base sessile and more or less auriculate; margins entire; apex attenuate; petiole absent or < 0.1 cm long; minor leaves 3–5 cm long, 1–2 cm wide, differing from the majors only in size. Inflorescence 0.1–0.3 cm long, opposite the leaves, unbranched, with 4–7 flowers, glabrous; peduncle < 0.1 cm long; pedicels *ca.* 0.8 cm long, 0.5 mm in diameter at the base and apex, filiform, nodding at anthesis, glabrous, articulated at the base; pedicel scars tightly packed and almost overlapping. Buds ellipsoid to rounded, the corolla exserted *ca.* halfway from the calyx tube just before anthesis. Flowers 5-merous, perfect. Calyx tube 1.5–2 mm long, conical, the lobes 2–3 mm long, *ca.* 1 mm wide, narrowly deltate to long-triangular with a 1–1.5 mm long projection that in live plants is a fleshy knob, glabrous. Corolla 0.8–1 cm in diameter, white, stellate, lobed ½ to 2/3 of the way to the base, the lobes *ca.* 0.4 cm long, 0.2 cm wide, planar at anthesis, minutely puberlent at the tips and along margins. Stamens 3–4 mm long; filament tube *ca.* 0.5 mm long, the free portion of the filaments <0.5 mm long, glabrous; anthers 2.5–3.5 mm long, *ca.* 1 mm wide, ellipsoid, yellow, poricidal at the tips, the pores elongating to longitudinal slits with age. Ovary glabrous; style 4–5 mm long, glabrous; stigma minutely capitate, the surface minutely papillose. Fruit a globose or depressed globose berry, *ca.* 1 cm in diameter, green or pale whitish green, glabrous, the pericarp thick, not markedly shiny; fruiting pedicels *ca.* 1.5 cm long, *ca.* 3 mm in diameter at the apex, woody, deflexed; calyx lobes in fruit persistent and slightly elongating, occasionally breaking off but always with > 1 mm remnants. Seeds *ca.* 30 per berry, not known from mature fruit.

**Figure 1.**
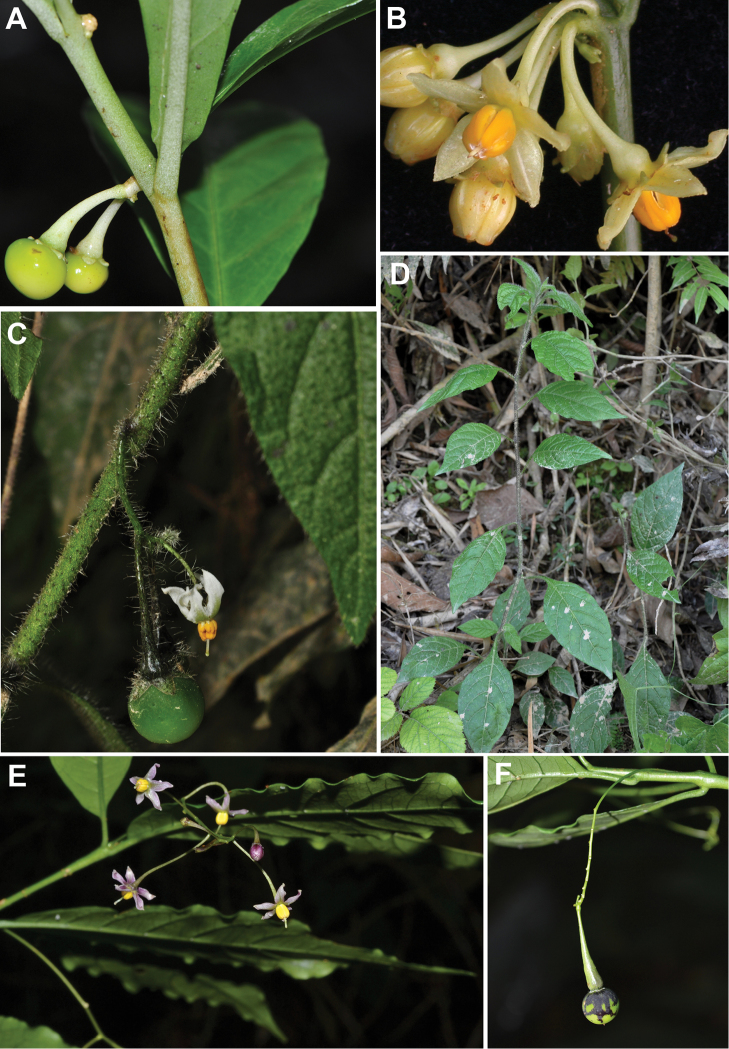
Photograph of living plants of *Solanum amorimii*, *Solanum apiahyense* and *Solanum filirhachis*. **A** Immature fruit of *Solanum amorimii* (*Giacomin et al.1962*) **B** Flowers of *Solanum amorimii* (*Amorim et al. 5210*) **C** Inflorescence with flower and fruit of *Solanum apiahyense* (*Giacomin et al. 1086*) **D** Habit of *Solanum apiahyense* (*Giacomin et al. 1086*) **E** Inflorescence, flower and leaves of *Solanum filirhachis* (*Giacomin et al. 1854*) **F** Fruit (immature) of *Solanum filirhachis* (*Giacomin et al. 1854*). Photographs: **A** (S. Knapp), **B** (A.M. Amorim), **C–F** (L.L. Giacomin).

**Figure 2.**
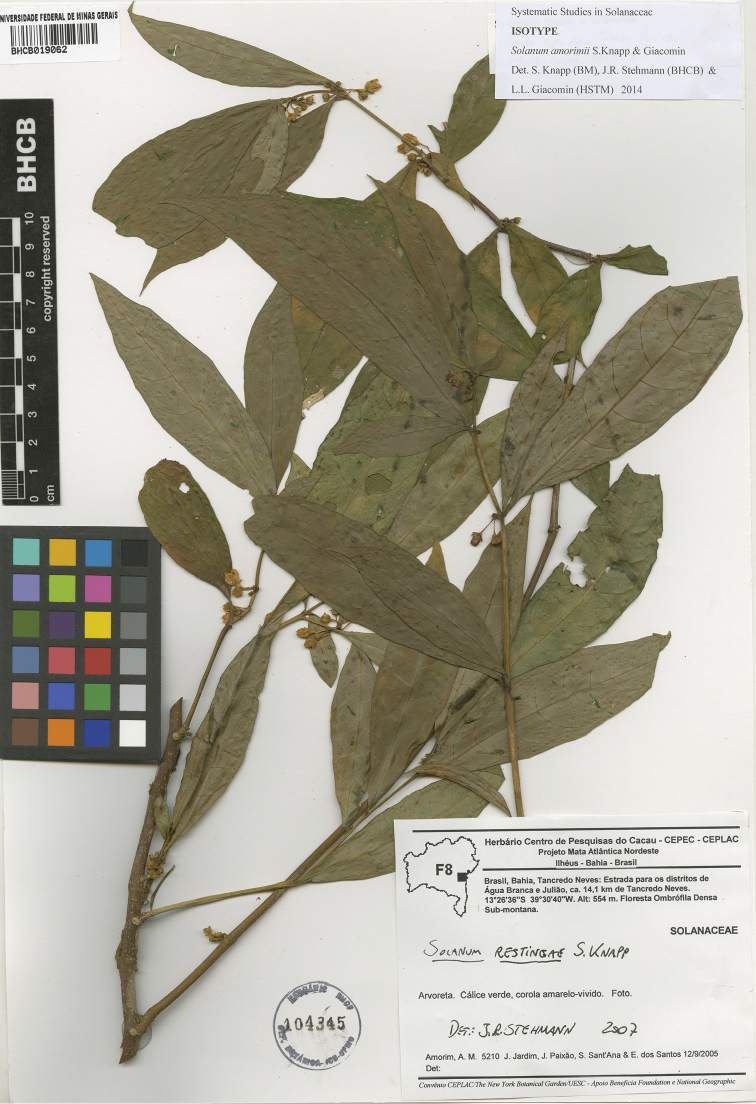
Isotype specimen of *Solanum amorimii* (*Amorim et al. 5210*, BHCB).

#### Distribution.

**Figure 3.**
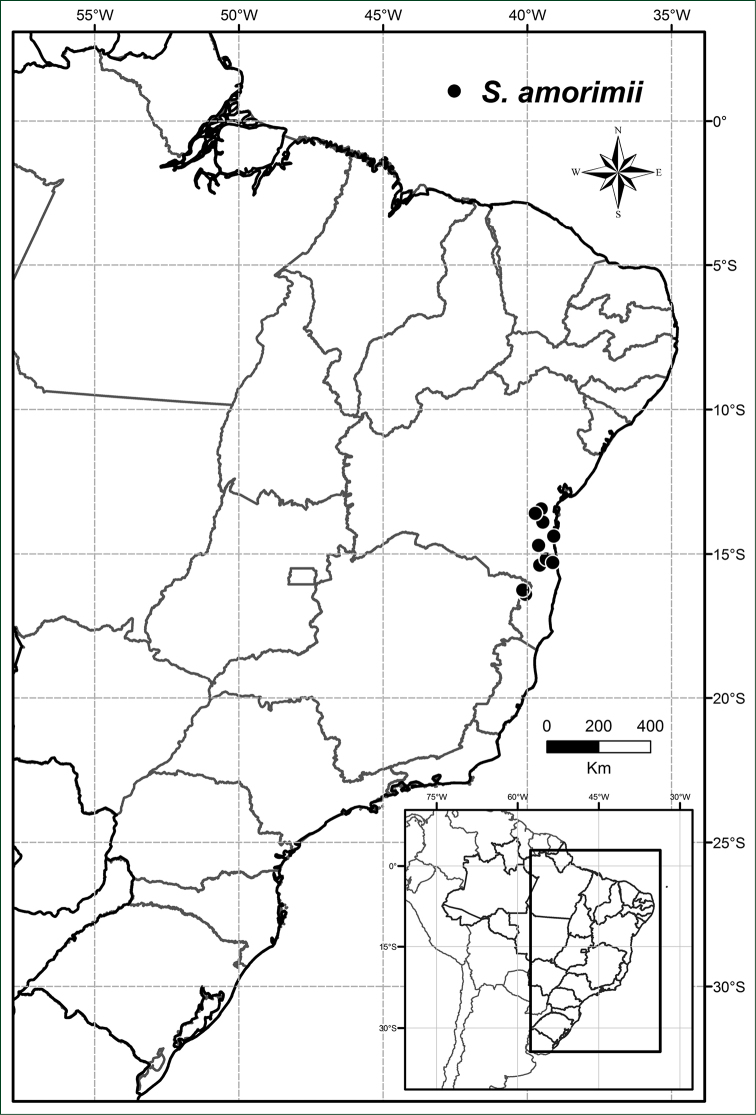
Distribution of *Solanum amorimii*.

Endemic to eastern Brazil in the states of Minas Gerais and Bahia, known from northernmost Minas Gerais and southern Bahia (Figure 3).

#### Ecology.

*Solanum amorimii* is found in the understory of wet Atlantic forests (*Floresta Ombrófila Densa*, Mata Atlântica; IBGE 2012) from 50–1000 m, most commonly found at around 500–900 m elevation in very preserved sites.

#### Phenology.

Flowering specimens have been collected from July to October but appears to peak in August; fruiting specimens have been collected from September to April.

#### Etymology.

The species epithet honours André M. Amorim, curator of the herbarium at CEPEC in Ilhéus, Bahia, and collector of the type specimen, whose knowledge of the flora of Bahia has helped many botanists in the region and beyond.

#### Preliminary conservation status (IUCN 2014).

Near-threatened (NT) B1, 2a, b (ii, iii); EOO 20,663 km^2^ (NT); AOO 40 km^2^ (EN). Although the large extent of occurrence (> 20,000 km^2^) places *Solanum amorimii* out of the vulnerable category, the small number of locations (5–10) and the fragmentation of its forest habitat mean it is of some conservation concern. Populations occur within several private protected areas (in Minas Gerais the only population is within a private reserve) so the species is afforded some protection. On the other hand, the known collections suggest the species is restricted to pristine sites, which are becoming increasingly rare. As with all Geminata species, it is possible that more populations remain to be collected; these plants are inconspicuous in the deep forest understory and usually occur in small, sparsely distributed populations.

#### Notes.

*Solanum amorimii* is morphologically very similar to the sympatric *Solanum restingae*, but can be distinguished by its much smaller flowers with long-triangular calyx lobes and by its unwinged stem. Both species grow in the understory of mostly undisturbed forests and can be small shrubs or treelets. *Solanum restingae* has markedly cucullate corolla lobes, and the calyx lobes are so small as to be almost non-existent, especially in fruit. Bud shape also differs between the two species, with those of *Solanum amorimii* being globose to somewhat ellipsoid and those of *Solanum restingae* more elongate with a distinct “nipple” from the cucullate corolla tips. In fruit the two species can be difficult to distinguish, but the winged stems of *Solanum restingae* and the presence of calyx lobes in *Solanum amorimii* should enable identification.

Leaves of *Solanum amorimii* are usually somewhat auriculate at the base, with the base not surrounding the stem but enlarged to a very short petiole. Plants grow in forest understory, sometimes in open places such as treefall gaps. From overall morphology this species would belong to the *Solanum arboreum* species group of Knapp (2002a), but its relationships have not been tested using molecular sequences.

#### Specimens examined.

**BRAZIL. Bahia:** Mun. Arataca, RPPN Caminho das Pedras, Serra do Peito-de-Moça, entrada a 9.5 km no Assent. Santo Antonio, mais 8.9 km ate a sede da RPPN, trilha de acesso ao topo da serra, após a Mormaço, 15°10'27"S, 38°20'22"W, 900–936 m, 26 Nov 2006 (fr), *A.M. Amorim et al. 6608* (CEPEC); Mun. Arataca, Serra do Peito-de Moça, estrada que liga Arataca a Una, ramal ca 22.4 km de Arataca com entrada do Assentiamento Santo Antonio, RPPN Caminho das Pedras, 15°10'25"S, 39°20'30"W, 1000 m, 20 Jan 2007 (fr), *A.M. Amorim et al. 6730* (CEPEC); Mun. Arataca, Serra do Peito-de Moça, RPPN do IESB, rodovia Arataca/Una, entrada a 9.5 km de cidade, mais 8.9 km de entrada, trilha do mormaço, 15°10'27"S, 39°20'22"W, 700–900 m, 12 Aug 2009 (fl), *L. Daneu et al. 81* (CEPEC); Mun. Arataca, Serra Novo Javi, RPPN do IESB, rodovia Arataca/Una, entrada a 9.5 km N, mais 8.9 km até a sede da RPPN, trilha da Serra, acesso *ca.* 1.5 km NE da sede, Topo da Serra, 15°10'42"S, 39°20'09"W, 12 Sep 2009 (fl), *L. Daneu et al. 96* (CEPEC); Mun. Arataca, Serra Novo Javi, RPPN do IESB, rod. Una/Arataca, entrada 9.5 km N, mais 8.9 km até a sede da RPPN, trilha da serra, acesso *ca.* 1.5 km NE da sede, topo da serra, 15°10'42"S, 39°20'09"W, 759 m, 12 Sep 2009 (fl), *L. Daneu et al. 121* (CEPEC); Mun. Camacan, RPPN Serra Bonita, trilha da pousada, 15°23'26"S, 39°33'55"W, 835–1000 m, 25 Aug 2007 (fl), *F.M. Ferreira et al. 1326* (CEPEC); Mun. Arataca, Serra do Peito-de Moça, Serra do Peito-de Moça-Serra das Lontras, estrada Arataca-Una, ramal 22.4 km de Arataca, assentamento Sto. Antonio, RPPN Caminho das Pedras, 15°10'25"S, 39°20'30"W, 1000 m, 23 Sep 2007 (fl), *F.M. Ferreira et al. 1452* (CEPEC); Reserva Pratigi, 28 km de Itamarati, 6 km no ramal a direita, sentido Gandu, 13°53'52"S, 39°27'26"W, 670 m, 22 Oct 2007 (fl), *F.M. Ferreira et al. 1563* (CEPEC); Mun. Uruçuca, estrada de Itacaré para Serra grande, pouco após km 43, ramal à direta após acesso para a cachoeira do Tijuipe, área explorada do plano de manejo, 14°23'12"S, 39°04'45"W, 4 Apr 2004 (fr), *P. Fiaschi et al. 2249* (CEPEC); Mun. Arataca, Serra Novo Javi, RPPN do IESB, Rod. Una/Arataca, entrada 9.5 km N, mais 8.9 km até a sede da RPPN, trilha da Serra acesso *ca.* 1.5 km NE do sede, Topo da Serra, 15°10'42"S, 39°20'09"W, 759 m, 12 Oct 2008 (fr), *J.G. Jardim et al. 5408* (CEPEC); Mun. Una, Rodovia BA-265, a 23 km de Una, 50–75 m, 26 Feb 1978 (fr), *S.A. Mori et al. 9299* (CEPEC, MO, NY); Mun. Almadina, Serra do Concavado, Rod. Almadina/Coaraci, *ca.* 5 km, 14°42'13"S, 39°36'09"W, 300 m, 19 Mar 2006 (fr), *J.L. Paixão et al. 838* (CEPEC);. Mun. Wenceslau Guimarães, *ca.* 3 km W of Nova Esperança, W edge of Reserva Wenceslau Guimarães, 13°36’ S, 39°43’ W, 500–600 m, 14 May 1992 (fr), *W.W. Thomas et al. 9244* (CEPEC, MO, NY, RB); Mun. Camacan, RPPN Serra Bonita, 9.6 km NNW of Camacan on road to Jacaraci and Jussari, then 6 km up road to Serra Bonita, 820 m, 21 Sep 2004 (fr), *W.W. Thomas et al. 14224* (NY). **Minas Gerais:** Mun. Santa Maria do Salto, Distrito de Talismã, RPPN Loredano Aleixo (Fazenda Duas Barras), 16°24'01"S, 40°03'24"W, 873 m, 31 Oct 2013 (fl, fr), *L.L. Giacomin et al. 1962* (BHCB, BM, UT); Mun. Santa Maria do Salto, RPPN Duas Barras, *ca.* 27 km do distrito de Talismã, trilha em direçåo a divisa com a Bahia, 16°14'56"S, 40°08'58"W, 8 Sep 2008 (fl), *R.P. Oliveira et al. 1636* (HUEFS).

### Solanum apiahyense

Taxon classificationPlantaeSolanalesSolanaceae

Witasek, Denkschr. Kaiserl. Akad. Wiss., Wien Math.-Naturwiss. Kl.79: 343. 1910.

Figures 1C, D , 4

#### Type.

Brazil. São Paulo. Apiahy, Feb 1891(fl), *J.I. Puiggari 3711* (lectotype, designated here: WU [WU0037965]).

#### Description.

Small erect shrubs, to 50 cm tall, often rhizomatous with a horizontal woody branch bearing several adventitious roots; young stems moderate to densely pubescent, with 4–8-celled hyaline trichomes to 2 mm long; new growth drying dark, densely pubescent; bark of older stems pale gray, glabrescent, not exfoliating. Sympodial units 3-plurifoliate, normally not geminate, if geminate, with leaves differing only in size. Leaves simple, 3.4–11 × 0.8–4 cm, elliptic to narrowly elliptic, membranous, slightly discolorous, shiny green adaxially when fresh, drying pale green beneath, dark above, not shiny, both surfaces moderate to densely pubescent with hyaline simple uniseriate trichomes 1–2 mm long with up to 5 cells, sometimes with a multicellular base (but see comments); primary veins 5–7 pairs, the midrib and primary veins darker abaxially, raised; base attenuate to acute, slightly decurrent onto the petiole, mostly symmetric; margins entire, not revolute, ciliate with antrorse hyaline trichomes; apex attenuate to acuminate; petioles 2.5–15 mm long, densely pubescent, with trichomes like those of the stems and leaves. Inflorescences 1.7 to 3.3 cm long, mostly lateral or less often strictly opposite the leaves, unbranched, with 3–5 flowers, moderate to densely pubescent, with hyaline trichomes like those of the stems and leaves; peduncle 4–15 mm long; pedicels 5 to 11 mm long, articulated at base; pedicel scars closely spaced *ca.* 1 mm apart. Buds globose to slightly elongate, the corolla mostly included in the calyx tube, exserted only just before anthesis. Flowers all perfect, 5-merous. Calyx tube up to 1 mm long, conical, getting reflexed, the lobes up to 0.9 mm long in flower, to 1.7 mm long in fruit, approximately 1.6 mm wide, acuminate and discretely keeled, adaxially, glabrous or papillose, covered with tiny 1–2-celled glandular trichomes, abaxially densely pubescent, with trichomes as those of the stem, or sometimes even longer, with 2.5 mm, and normally 5–6 cells. Corolla 1.5–1.7 cm in diameter, white, stellate, membranous, lobed from 2/3 to 3/4 of the way to the base, the lobes 7.5–9 mm long, 3–3.5 mm wide, reflexed at anthesis, deltate to lanceolate, glabrescent adaxially, abaxially sparsely pubescent, with 3–4-celled delicate simple trichomes of *ca.* 0.5 mm along the midvein, with tufts of few celled tiny trichomes less than 0.1 mm long on the tips and margins. Stamens 3.2–3.6 mm long; filament tube *ca.* 0.5 mm long, the free portion of the filaments up to 0.6 mm long equal in length or slightly unequal, and when so, one filament slightly longer (barely visible in dried material), glabrous; anthers 2.6–2.8 mm long, 1.6–1.8 mm wide, ellipsoid, slightly connivent, yellow, slightly sagittate at the base, the pores directed introrsely, opening into longitudinal slits at maturity. Ovary glabrous; style 4.2–5 mm long, white, straight, glabrous; stigma capitate, light green. Fruit a globose berry 0.7–1.4 cm in diameter (immature?), dull green, drying dark, the pericarp glabrous and not markedly shiny; fruiting pedicels 1.2–2 cm long, *ca.* 0.7 mm in diam. at the base, to 1.1 mm at the apex, with a slight constriction at the receptacle; calyx lobes in fruit somewhat enlarged. Seeds approximately 70 per fruit, known only from very young fruits, possibly flattened and with a marginal wing when fully developed.

**Figure 4.**
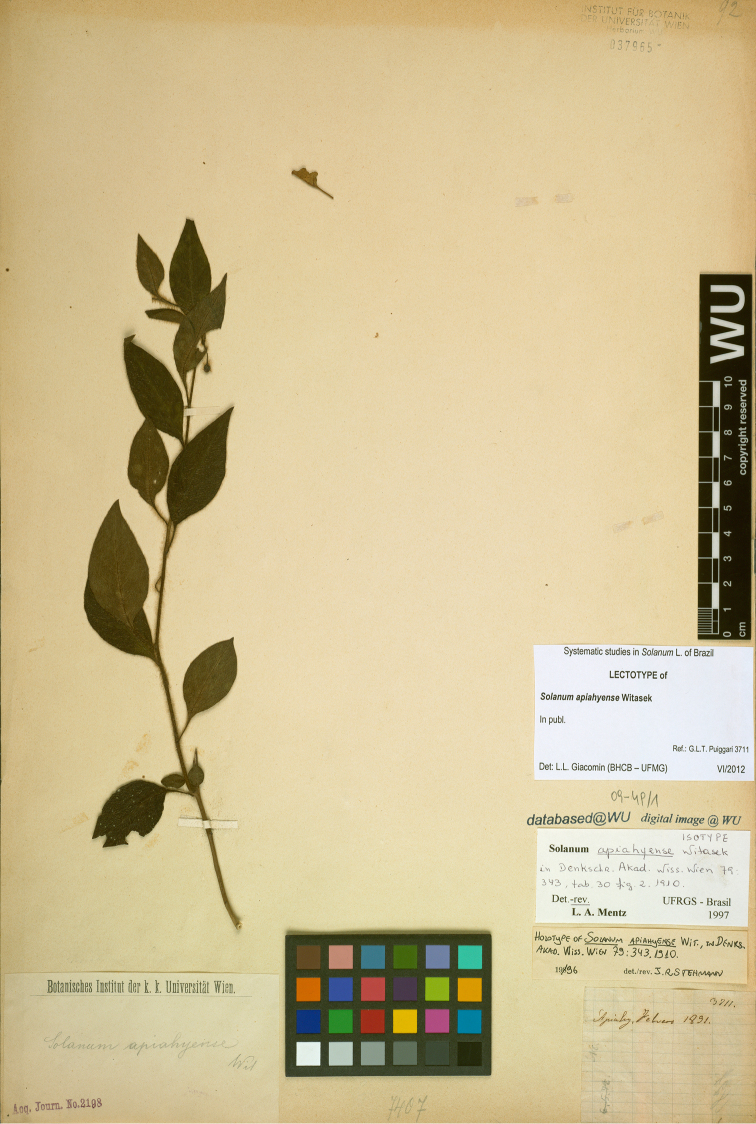
Lectotype specimen of *Solanum apiahyense* (*Puiggari s. n.*, WU). Reproduced with permission of the University of Vienna.

#### Distribution.

**Figure 5.**
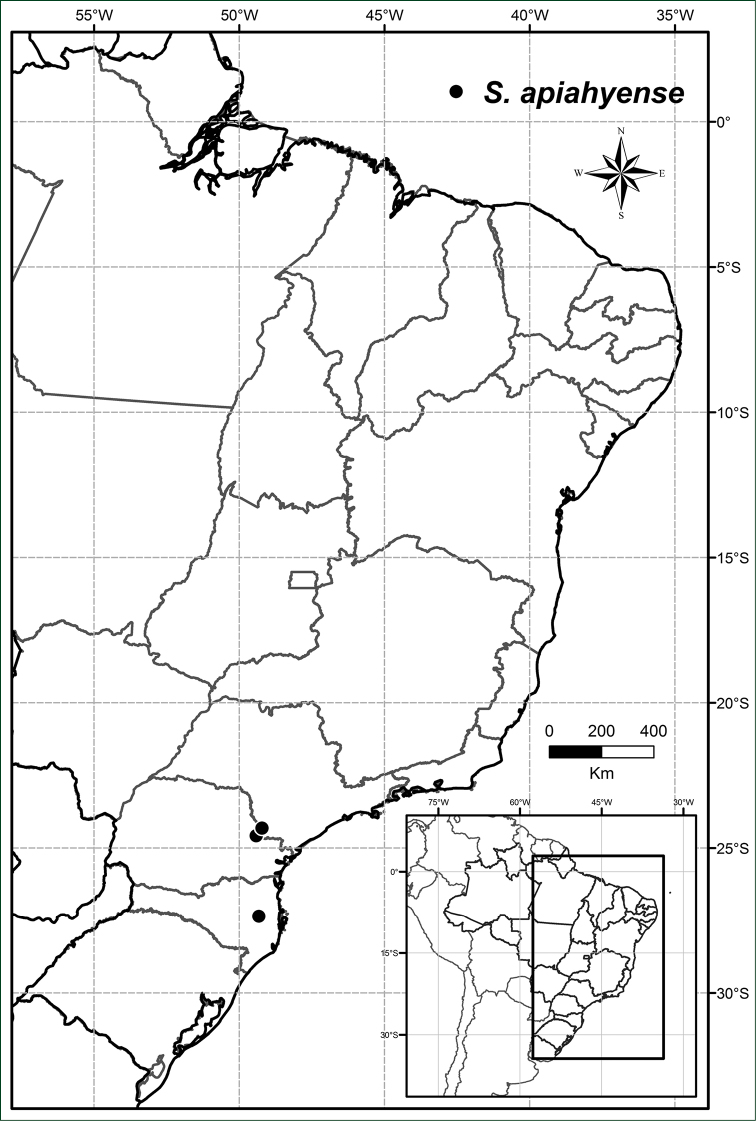
Distribution of *Solanum apiahyense*.

In the Serra do Mar mountain range in the Brazilian states of Paraná, Santa Catarina and São Paulo (Figure 5).

#### Ecology.

*Solanum apiahyense* is a rare and inconspicuous shrub of the understory and edges of well preserved and secondary fragments of the montane Brazilian Atlantic rainforest (*Floresta Ombrófila Densa* of IBGE 2012; Mata Atlântica), from 600 to 900 m. Although most collections are from well preserved sites, *Solanum apiahyense* is not exclusively associated with shaded environments. The species is also found along unpaved roadsides close to the type locality.

#### Phenology.

Fertile specimens are known from September to February. Mature fruits were observed only in October.

#### Etymology.

The epithet refers to the type locality, the city of Apiaí in southern São Paulo state.

#### Preliminary conservation status (IUCN 2014).

Endangered (EN) B1; B2 ab (ii, iii, iv). EOO 3,208 km^2^ (EN); AOO 16 km^2^ (EN). Although the species occurs in a wide latitudinal range, it is locally rare, and is known from only six localities. None of the known populations are from within protected areas.

#### Notes.

*Solanum apiahyense*, described more than a century ago (Witasek 1910), has not been assigned to any infraspecific group of *Solanum* so far. Recent phylogenetic analysis using molecular data (Giacomin 2015) has shown it to be closely related to *Solanum trachytrichium*, which was previously assigned to the Geminata clade (Knapp 2002a, 2008) and to its own subsection when originally described (subsect. *Silicosolanum* Bitter; Bitter 1919). Bitter (1919) based this on the unusual trichome morphology of hooked cells arising from a flattened multicellular base that give the leaves a feeling of sandpaper in herbarium specimens. Although molecular data support a close relationship between *Solanum apiahyense* and *Solanum trachytrichium*, the affinities of this clade are not clear-cut. Data from combined markers place it as sister to all other Geminata clade species, but with low support. In analyses of individual markers, it emerges as sister to either the Brevantherum or Geminata clades depending upon the marker used (Giacomin 2015).

Morphologically both taxa are easy to distinguish from most other Geminata species, and have the following assemblage of characters: both are small shrubs with leaves mostly not geminate, they have leaf trichomes with an expanded multicellular base and relatively large flowers (>1.5 cm in diameter). Among them, *Solanum apiahyense* and *Solanum trachytrichium* are easy to distinguish: *Solanum trachytrichium* has a unique scabrous indumentum on the leaf surfaces and stems, composed of short unicellular hooked trichomes on a mound-like multicellular base, while in *Solanum apiahyense* the surface is not rough to the touch, and although some trichomes with multicellular bases can be seen on leaves, these are translucent, very long (*ca.* 2 mm) and mostly 5-7-celled. These long trichomes of *Solanum apiahyense* are easily seen on the new growth, while *Solanum trachytrichium* trichomes are not visible to the naked eye. In addition, the flowers of *Solanum apiahyense* are slightly smaller, 1.5–1.7 cm in diameter versus 1.6–2.2 cm in *Solanum trachytrichium*.

In the past, the epithet *Solanum apiahyense* has been applied to more than one species of the *Solanum inornatum* group (part of the Brevantherum clade; Giacomin and Stehmann 2014) by various *Solanum* taxonomists, although they are now known to not be closely related. Although members of the *Solanum inornatum* group (e.g., *Solanum inornatum* Witasek, *Solanum bradei* Giacomin & Stehmann and relatives) and *Solanum apiahyense* are similar in habit and in having pubescence of long, translucent trichomes, they can be readily distinguished by close examination of the trichomes; those of *Solanum apiahyense* are multicellular with 5-7(8) cells while those of members of the *Solanum inornatum* group are mostly 3-celled (probably representing modified stellate hairs, Giacomin and Stehmann 2014). Fruiting specimens of *Solanum apiahyense* have peduncles longer than 1 cm and the pedicels are strongly apically expanded and constricted just beneath the calyx lobes (see Figure 1C), while in the species of the *Solanum inornatum* group species, the peduncles do not exceed 1 cm and the pedicels are never apically expanded with a distal constriction. Examination of trichomes with a 10× hand lens will allow easy identification of both flowering and fruiting material.

The type material found at WU (*Puiggari 3711*) consists of a single sheet, and does not match the photograph of a dried specimen in the original publication (Witasek 1910: tab. 30, fig. 2). It should therefore be treated as an isotype (Mentz and Oliveira 2004). As no further material could be found in other possible herbaria where J.I. Puiggari deposited his collections, the specimen at WU is here designated as a lectotype.

#### Specimens examined.

**BRAZIL. Paraná:** Mun. Cerro Azul, Serra Paranapiacaba, 20 Nov 1970 (fl), *G. Hatschbach & O. Guimarães 25528* (MBM, RB); Mun. Doutor Ulysses, Barra do Teixeira, 16 Sep 2006 (fl, fr), *J.M. Silva* (HUFU, MBM, RB). **Santa Catarina:** Mun. Vidal Ramos, Mina Bugre, 27°21'35"S, 49°19'12"W, 598 m, 22 Sep 2009 (fl, fr), *A. Korte & A. Kniess 243* (BHCB, FURB). **São Paulo:** Mun. Bom Sucesso de Itararé, Estrada de terra para Bom Suceso de Itararé, Próximo a Mineração de ouro São Judas (3 km após), 24°19'13.19"S, 49°12'49.49"W, 891 m, 11 Oct 2009 (fl, fr), *L.L. Giacomin et al. 1097* (BHCB, BM, NY, RB); Mun. Bom Sucesso de Itararé, Estrada Bom Sucesso de Itararé, 2 km antes da Mineração São Judas, 24°19'13"S, 49°13'04"W, 15 Dec 1997 (fr), *J.M. Torenzan et al. 647* (IAC, ESA, FUEL, SPSF, UEC).

### Solanum filirhachis

Taxon classificationPlantaeSolanalesSolanaceae

Giacomin & Stehmann sp. nov.

urn:lsid:ipni.org:names:77145588-1

Figures 1E, F , 6

#### Diagnosis.

Differs from the sympatric *Solanum campaniforme* Roem. & Schultes in its deep forest habitat, leaves with ruffled margins, flowers less than 1 cm in diameter, pedicels with a constriction at the distal end that are swollen in fruit, and few seeds.

#### Type.

Brazil. Espírito Santo: Mun. Santa Teresa, Comunidade de Santo Antônio, Propriedade do Sr. Boza, fragmento de floresta ombrófila densa após plantação de eucalipto, à direita da entrada, descendo o vale, 19°54'32"S, 40°35'26"W, 740 m, 8 Jun 2012 (fl, fr), *L.L. Giacomin, L. Bohs, Y.F. Gouvêa & F.Z. Saiter 1854* (holotype: BHCB [2 sheet holotype: sheet 1 (fl) BHCB019056; sheet 2 (fr) BHCB019057]; isotypes: BM, MBML, NY, RB).

#### Description.

Erect shrubs to small trees, up to 3 m tall, normally branching close to the apex, the upper stems ascendant; young stems terete, glabrous; new growth brownish, glabrous. Bark of older stems turning pale greyish brown, glabrous, not exfoliating. Sympodial units difoliate, mostly geminate, with leaves not differing in shape or size. Leaves simple, 4.6–15.9 cm long, 1.3–4.9 cm wide, narrowly elliptic, membranous to chartaceous, slightly discolorous when dry, the adaxial surface glabrous, dark green and somewhat shiny in live plants, the abaxial surface sparsely pubescent with simple uniseriate 7–12-celled trichomes to 1 mm long in tufts in the primary vein axils, occasionally extending to the midrib; primary veins 5–9 pairs, yellowish green, discretely raised above, raised beneath; base attenuate to acute, slightly decurrent onto the petiole, sometimes asymmetric; margins entire, slightly undulate (ruffled) and revolute, apex long-attenuate to acuminate; petioles 1–9 mm long, glabrous. Inflorescences 3.5 to 26 cm long, opposite the leaves or internodal, unbranched, slender and very delicate, with 18–60 flowers, but bearing normally with 4–10 flowers at a time, glabrous; peduncle 1.8–3.8 cm long; pedicels 7–18 mm long, *ca.* 0.4 mm in diam. at the base, *ca.* 0.9 mm in diameter at the apex, with a constriction at the receptacle, articulated at base, unevenly spaced 1.7 to 10 mm apart. Buds globose, the corolla completely exserted from the calyx tube before anthesis. Flowers all perfect, 5-merous. Calyx tube to 1 mm long, conical, the lobes *ca.* 0.2 mm long, *ca.* 1.5 mm wide, acuminate and somwewhat keeled, papillose adaxially, glabrous abaxially. Corolla 6–8 mm in diameter, normally whitish purple adaxially, light purple abaxially, stellate, membranous, lobed more than ¾ the way to the base, the lobes 4–5 mm long, 1–1.7 mm wide, spreading at anthesis and becoming reflexed in older flowers, deltate to lanceolate, glabrous on both surfaces, minutely papillose at tips and margins. Stamens 2.5–3 mm long; filament tube *ca.* 0.3 mm long, the free portion of the filaments up to 0.2 mm long, equal in length or slightly unequal, and when so, two filaments slightly longer (barely visible in dried material), glabrous; anthers 2–2.5 mm long, 1.2–1.5 mm wide, ellipsoid, slightly connivent, yellow, poricidal at the tips the pores directed introrsely, elongating to longitudinal slits with age. Ovary glabrous; style 4–6 mm long, white, straight, glabrous, the stigma light grayish green, capitate. Fruit a globose berry 1–1.5 cm in diameter, dull green at maturity, with irregular black spots (Figure 1F) drying grayish brown, the pericarp glabrous, not shiny; fruiting pedicels 2.0–2.4 cm long, clearly obconical, *ca.* 0.5 mm in diam. at the base, widening markedly towards the apex to *ca.* 2.5 mm in diam.; calyx lobes in fruit *ca.* 1.5 mm long, commonly broken off in dried fruiting material. Seeds 20–25 per berry, 2.5–4.5 mm long, 2–3.3 mm wide, ovoid-reniform to somewhat flattened towards the margins, light to dark brown, the surface irregularly pitted, the testal cells undulate.

**Figure 6.**
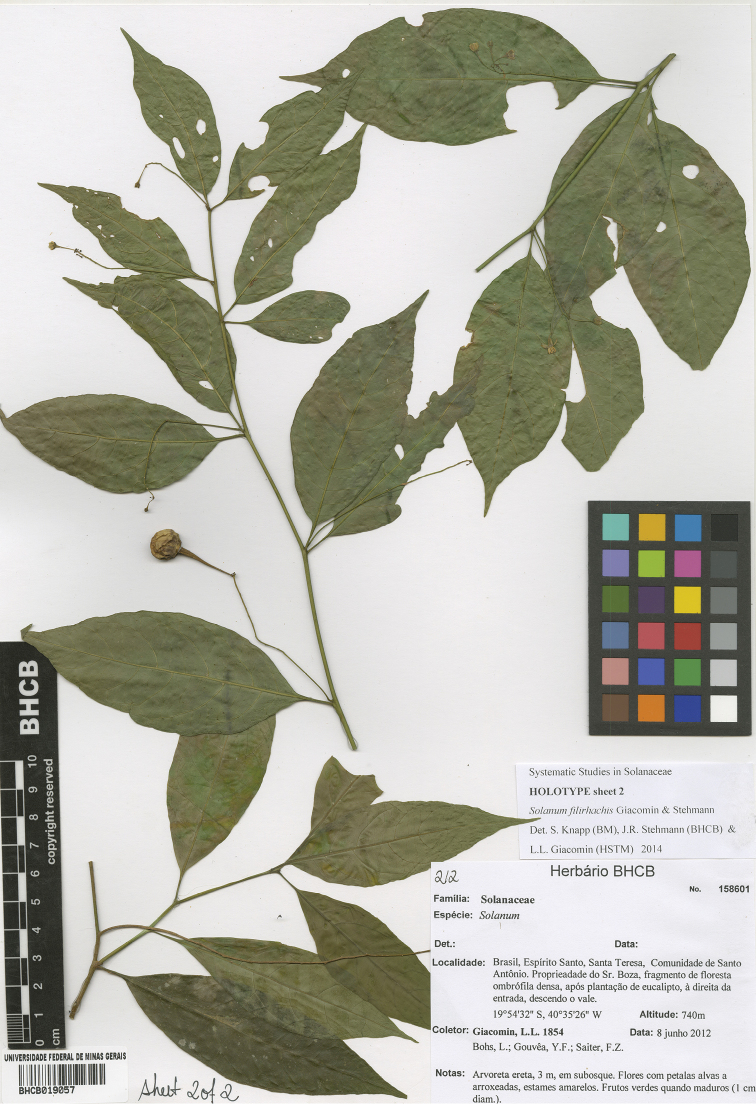
Holotype specimen (sheet two) of *Solanum filirhachis* (*Giacomin et al. 1854*, BHCB019057). Reproduced with permission of the Universidade Federal de Minas de Gerais.

#### Distribution.

**Figure 7.**
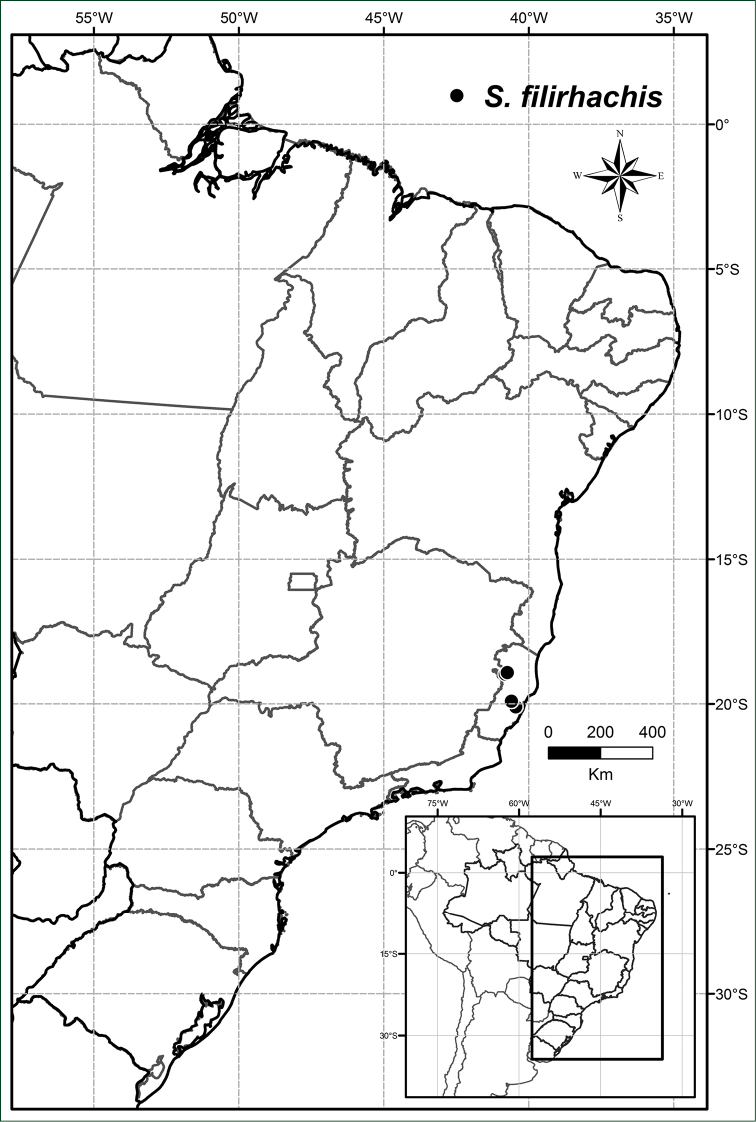
Distribution of *Solanum filirhachis*.

Restricted to the state of Espírito Santo (Figure 7), in south-eastern Brazil. Collections are known from the central and northern parts of the state, from both sides of the Rio Doce.

#### Ecology.

Rare in the understory of well-preserved fragments of the sub-montane and montane Brazilian Atlantic coastal rainforest (*Floresta Ombrófila Densa*; IBGE 2012), normally in formations where granitic outcrops are present or close by, in elevations ranging from 200 to 750 m.

#### Phenology.

Fertile specimens of *Solanum filirhachis* are known mostly from the rainy season (from November to March), but the type collection from June indicates that the species might be fertile for a longer period. Mature fruits were observed in specimens from November and June.

#### Etymology.

The epithet refers to the long and slender inflorescence rachis, which is not observed in any of the Brazilian sympatric species, although a common feature in some species of the *Solanum confine* group from Colombia, Ecuador and Venezuela (Knapp 2002a).

#### Preliminary conservation status (IUCN 2013).

Endangered (EN) B1, B2 ab (ii, iii, iv); EOO 1,136 km^2^ (EN); AOO 20 km^2^ (EN). *Solanum filirhachis* is currently known from only five localities, and all collections are from within private properties, where agriculture (both large and small scale) is known to occur. Despite the fact that it inhabits higher elevations that are usually harder to access and not always suitable for agriculture, we strongly recommend that further efforts to map new populations of the species should be undertaken, mainly within protected areas with similar forest types. Although the type locality of Santa Teresa in central Espirito Santo has several well preserved fragments of forest, the landscape has been rapidly transformed in the last few decades to *Eucalyptus* and coffee plantations, and summer vacation homes (cottages).

#### Notes.

*Solanum filirhachis* is remarkably similar to a suite of species of the Geminata clade with ruffled leaf margins (see Figure 1E) and long filiform inflorescences (*Solanum leptorhachis* Bitter and *Solanum nematorhachis* S.Knapp from the W Andean slopes in Colombia and Ecuador and *Solanum tenuiflagellatum* S.Knapp of Venezuela). Knapp (2002a, 2008) treated these as members of her *Solanum confine* species group, all of whose members have a thin inflorescence rhachis, small flowers and leaves with ruffled (undulate) margins, although this latter character is impossible to see in herbarium specimens. *Solanum filirhachis* differs from those species in its distribution and in the tufts of trichomes in the abaxial leaf vein axils (domatia); other members of this morphologically similar set of species are glabrous or have fine, golden pubescence. The only Brazilian species Knapp (2002) placed in this group was *Solanum stipulatum* which can be easily distinguished from *Solanum filirhachis* by its shorter inflorescences, flowers with reflexed corolla lobes and winged stems with anisophyllous difoliate, geminate sympodial units. *Solanum stipulatum* is usually a shrub of watercourses, and often grows amongst rocks and is submerged in floods, while *Solanum filirhachis* is a slender treelet of forest understory. The relationships of the *Solanum confine* group have not yet been tested using molecular markers.

Another Brazilian species with which *Solanum filirhachis* could be confused is *Solanum campaniforme* that has similar (but somewhat stouter) elongate inflorescences and tufts of uniseriate trichomes in the abaxial leaf vein axils. *Solanum filirhachis* has leaves with ruffled margins tht normally dry pale green and smaller flowers (0.6–0.8 cm in diameter) that (at least in the type specimen) are tinged purple; *Solanum campaniforme* has leaves with entire, non-ruffled margins that normally dry black or brownish black and larger flowers (1.2–1.8 cm in diameter) with strongly cucullate corolla lobes.

We have designated a two sheet holotype for *Solanum filirhachis* in order to represent both flower and fruit in the type sheets.

#### Specimens examined.

**BRAZIL. Espírito Santo:** Mun. Águia Branca, Assentamento 16 de Abril, 18°54'25"S, 40°44'05"W, 150-200 m, 15 Mar 2006 (fl), *V. Demuner et al. 1919* (MBML, BHCB); Mun. Santa Leopoldina, Colina Verde (Morro do Agudo), prop. Israel Elias Ramos (trilha da casa), 20°06'12"S, 40°26'30"W, 250-370 m, 29 Nov 2007 (fl, fr), *V. Demuner et al. 4628* (MBML, BHCB); Mun. Santa Leopoldina, Pedra Branca, mata na Serra Santa Lucia, prop. Cristiano Bremencampi, 20°01'36"S, 40°29'32"W, 300-600 m, 30 Nov 2007 (fr), *V. Demuner et al. 4655* (MBML, BHCB). Mun. Águia Branca, Rochedo, Trilha do Córrego, prop. Ailton Corteleti, 18°57'21"S, 40°48'05"W, 300-400 m, 19 Dec 2007 (fl, fr), *V. Demuner et al. 4817* (MBML, BHCB).

### Solanum lacteum

Taxon classificationPlantaeSolanalesSolanaceae

Vell., Fl. Flumin. 82. 1829 [“1825”].

**Figure 8.**
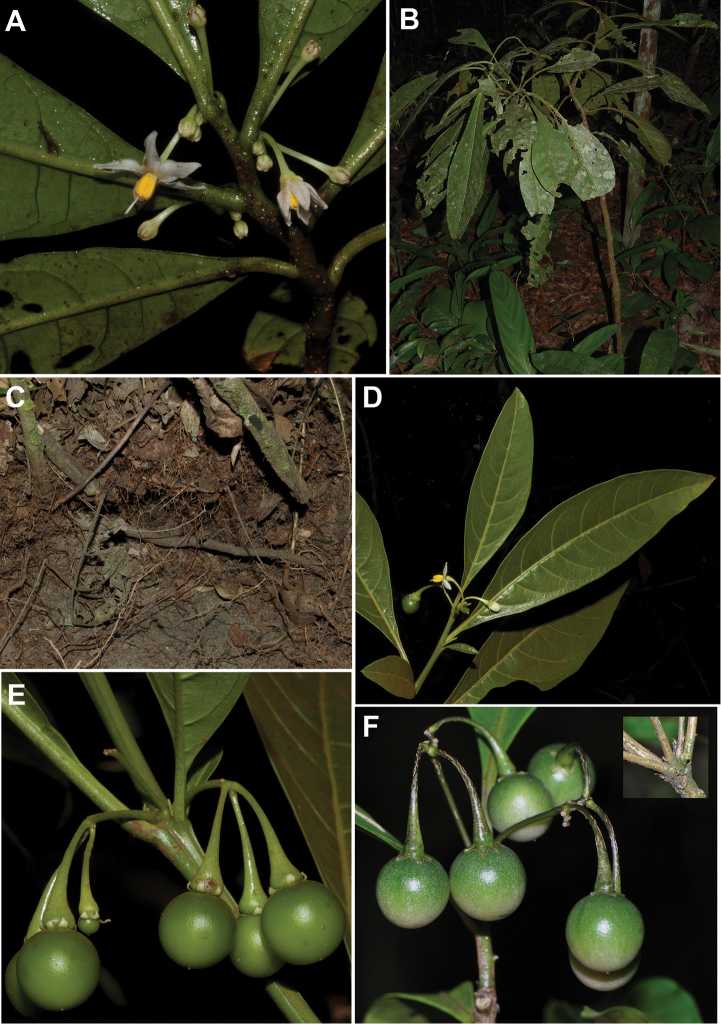
Photograph of living plants of *Solanum lacteum*, *Solanum psilophyllum* and *Solanum verticillatum*. **A** Inflorescence and flower of *Solanum lacteum* (*Agra et al. 7284*) **B** Habit of *Solanum lacteum* (from Linhares, ES; no voucher) **C** Habit of *Solanum psilophyllum* showing rhizomatous growth (*Giacomin et al. 186*) **D** Flowers and young stems of *Solanum psilophyllum* (*Giacomin et al. 186*) **E** Fruit of *Solanum psilophyllum* (*Giacomin et al. 186*) **F** Immature fruit of *Solanum verticillatum*, inset shows pseudo-verticillate branching pattern (*Giacomin et al. 2016*). Photographs: **A–E** (J.R. Stehmann), **F** (S. Knapp).

**Figure 9.**
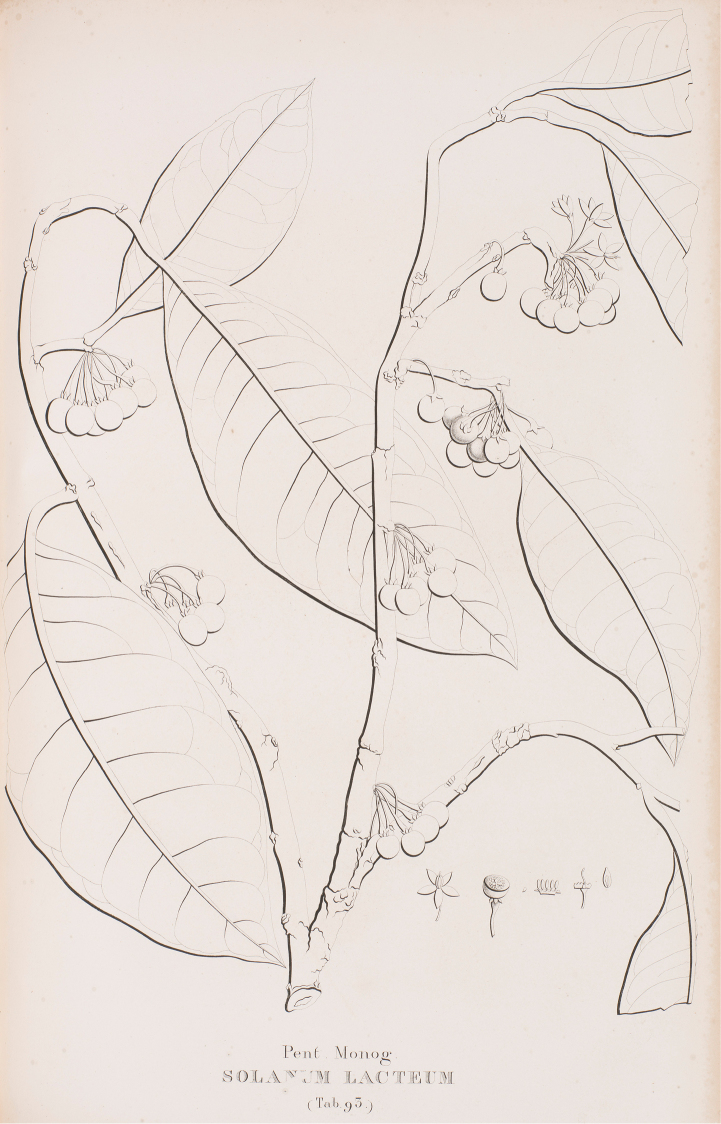
Lectotype of *Solanum lacteum*. Vellozo (1831) Volume 2, plate 93. Reproduced with permission of the Natural History Museum Library.

**Figure 10.**
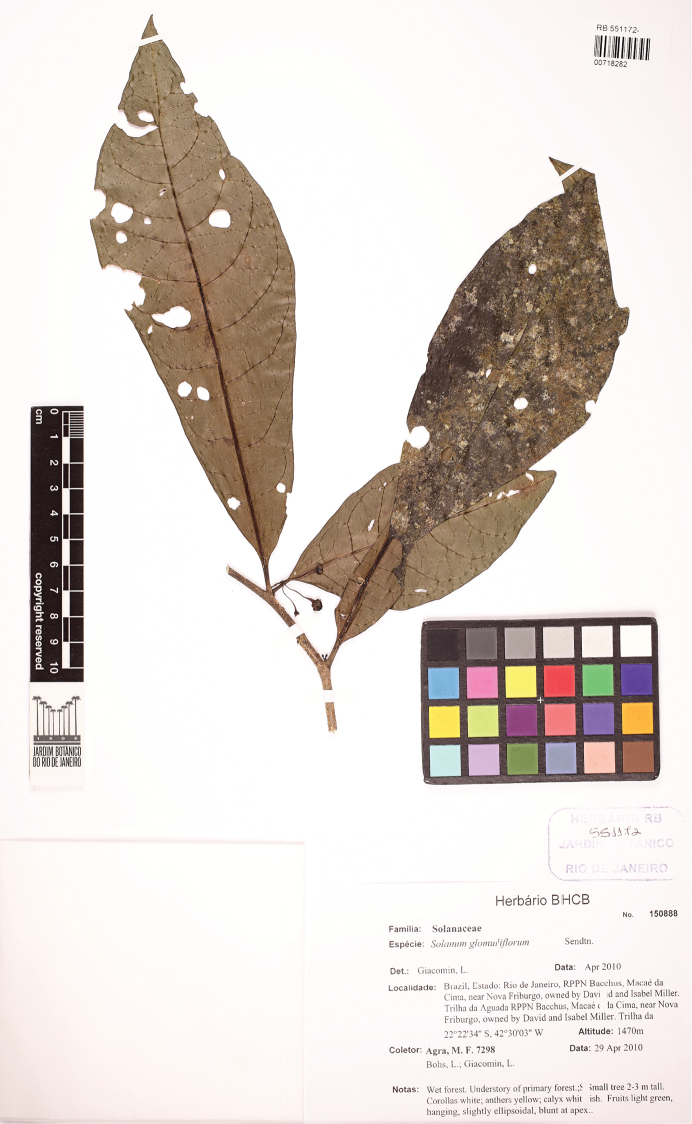
Epitype specimen of *Solanum lacteum* (*Agra et al. 7284*, RB). Reproduced with permission of the Jardim Botânico do Rio de Janeiro.

Figures 8A, B , 9 , 10

**Figure 11.**
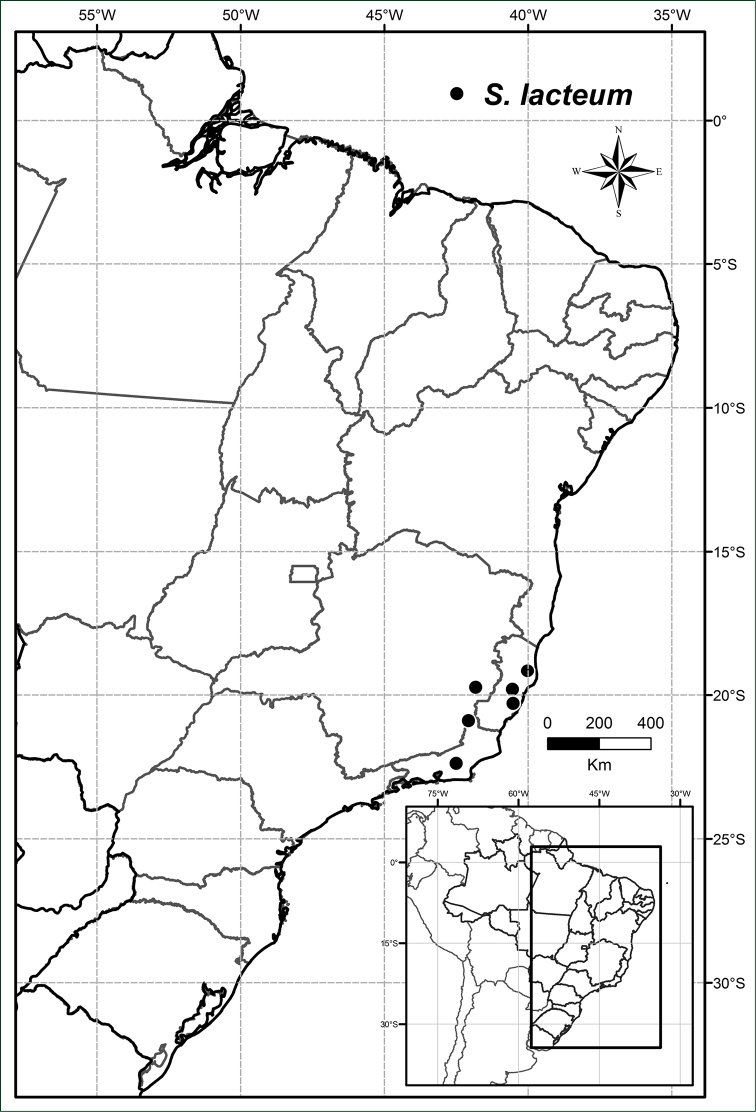
Distribution of *Solanum lacteum*.

**Figure 12.**
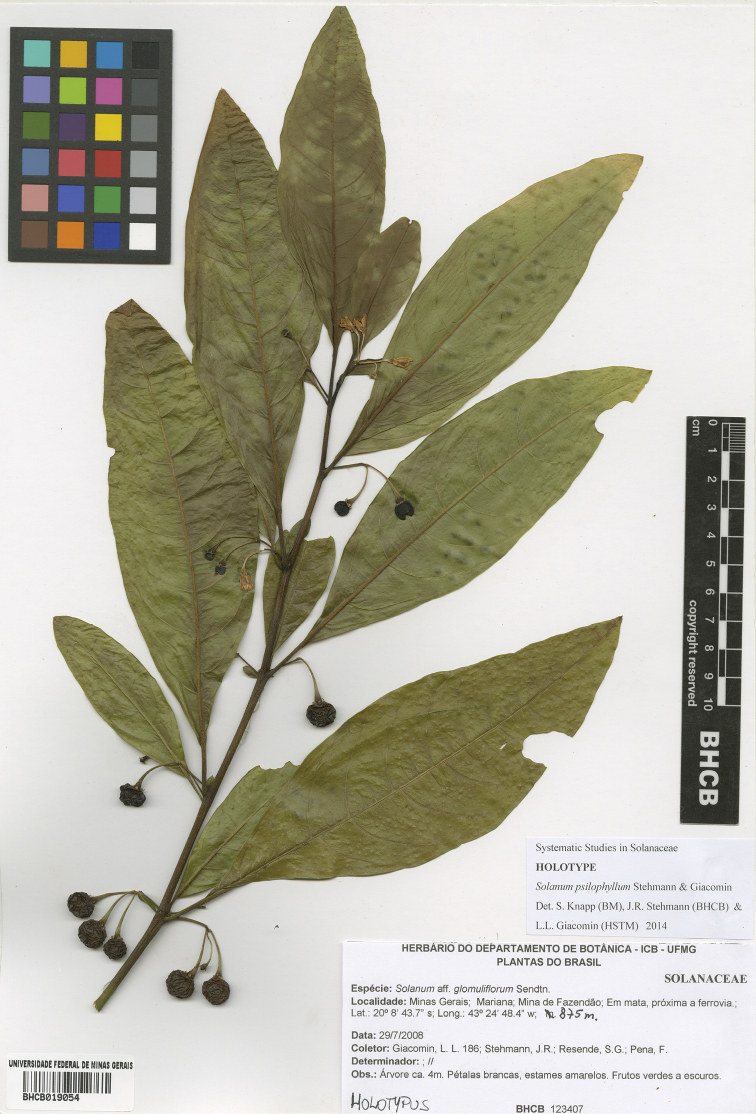
Holotype specimen of *Solanum psilophyllum* (*Giacomin et al. 186*, BHCB019054). Reproduced with permission of the Universidade Federal de Minas Gerais.

**Figure 13.**
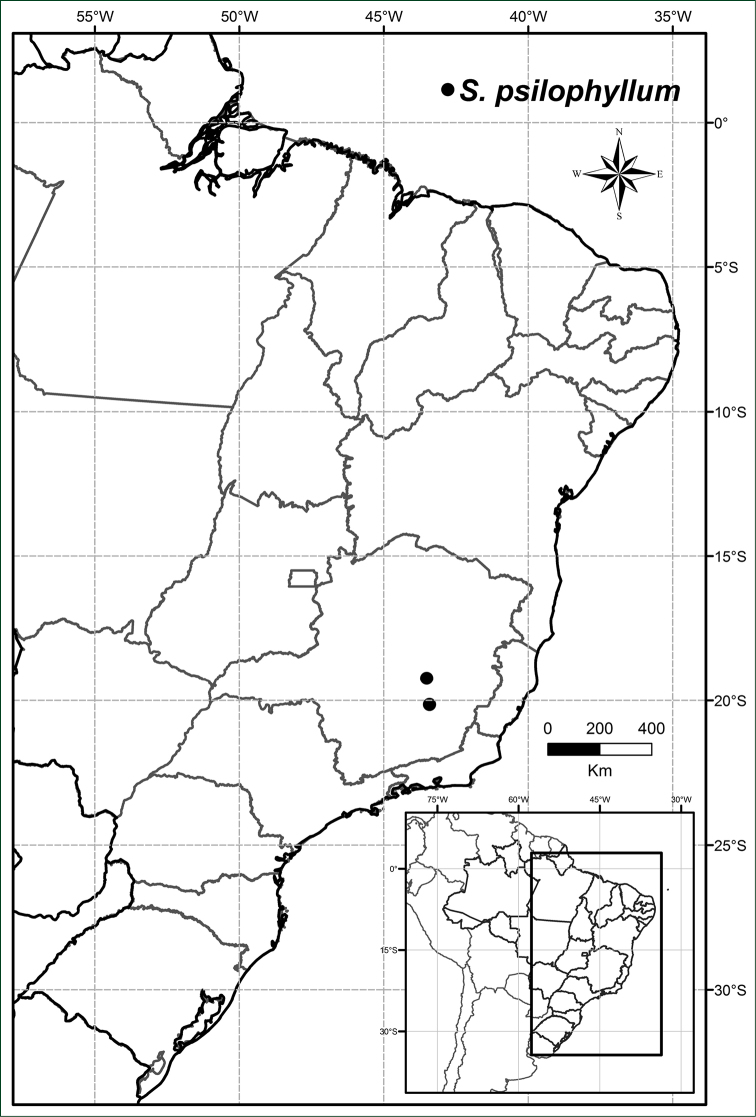
Distribution of *Solanum psilophyllum*.

**Figure 14.**
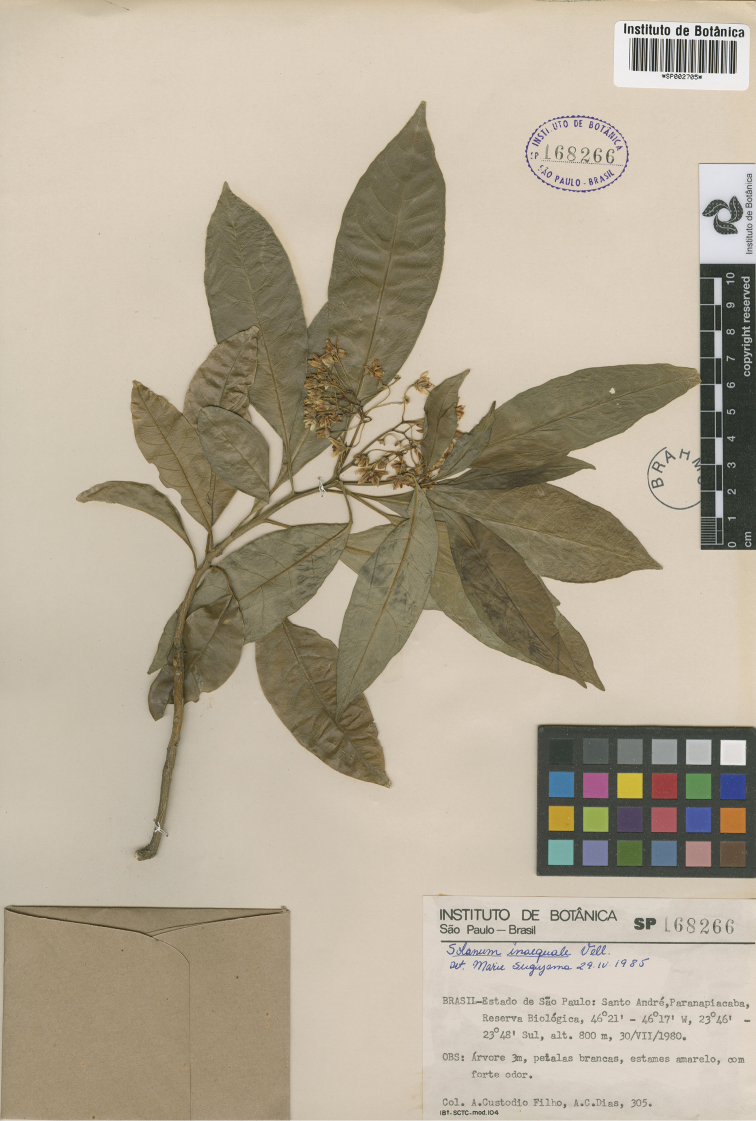
Holotype specimen of *Solanum verticillatum* (*Custodio Filho & Dias 305*, SP002705). Reproduced with permission of Instituto de Botânica, São Paulo.

**Figure 15.**
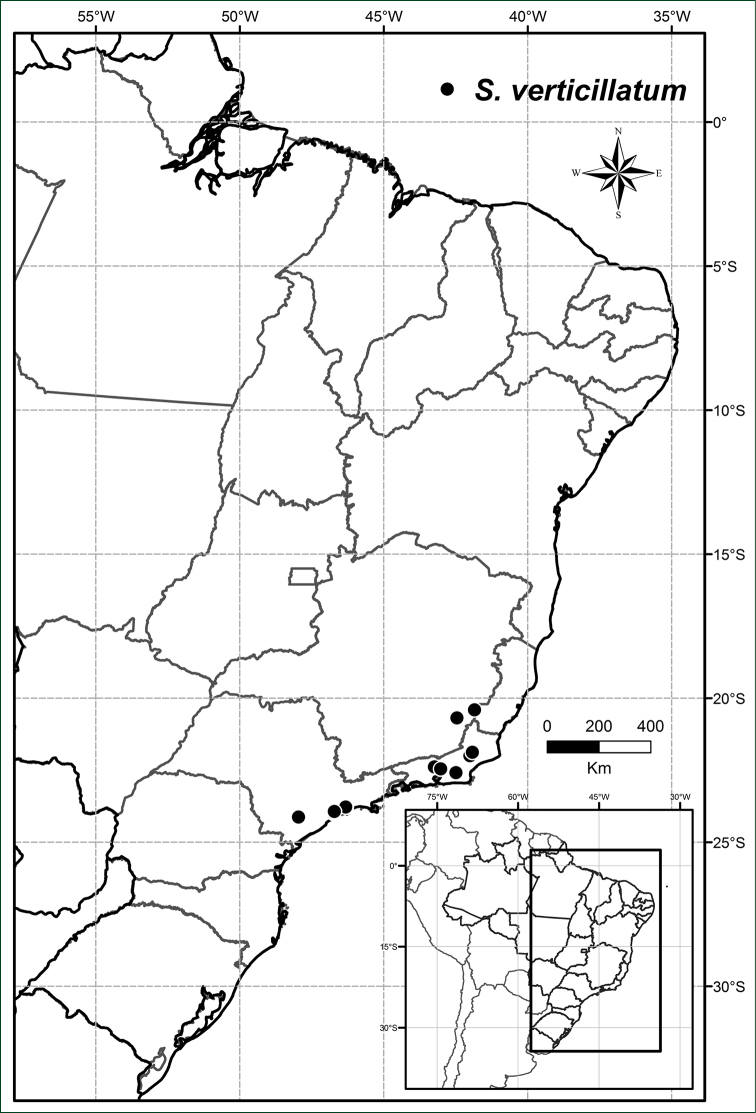
Distribution of *Solanum verticillatum*.

Solanum cormanthum Vell., Fl. Flumin. 86. 1829 [1825].
Solanum lacteum Type. Brazil Rio de Janeiro: “Praedii S. Crucis” (no specimens located; lectotype, designated here: Vellozo, Flora fluminensis icones 2: tab. 113. 1831).
Solanum glomuliflorum Sendtn., Fl. Bras. [Martius] 10: 24, tab 3, fig. 11–15. 1846.
Solanum lacteum Type. Brazil. Rio de Janeiro: “Serra d’Estrella” [Serra de Estrela] (fr), *H.W. Schott [5412] s.n.* (lectotype, designated here: F [F-874710]).

#### Type.

Brazil. Sin loc. [probably Rio de Janeiro] “Silvis nondum cultis ad rivulae, vel stagna crescit” (no specimens located; lectotype, designated here: Vellozo, Flora fluminensis icones 2: tab. 93. 1831; epitype, designated here: Brazil. Rio de Janeiro: Mun. Nova Friburgo, RPPN Bacchus, Macaé da Cima, near Nova Friburgo, owned by David and Isabel Miller, Trilha da Aguada, 22°23'34.4"S, 42°30'03.4"W, 1470 m, 29 Oct 2012 (fl, fr), *M.F. Agra, L. Bohs & L.L. Giacomin 7298* (RB [RB00718282, accession number 551172]; duplicates in BHCB, JPB, UT).

#### Description.

Shrub or small treelet 1–3 m (occasionally as small as 25–30 cm or as tall as 5 m); young stems terete, glabrous; new growth glabrous or minutely papillate; bark of older stems pale brown, with prominent paler lenticels. Sympodial units difoliate, geminate or more usually not geminate; leaves of a pair usually differing in size but not in shape. Leaves simple, 9.5–25 cm long, 3.5–9 cm wide, narrowly obovate, widest in the distal half, membranous, glabrous on both surfaces, the abaxial surface paler in dry specimens; primary veins 6–10 pairs, drying dark abaxially; base attenuate; margins entire; apex bluntly acute to attenuate; petiole 1–3 cm long, glabrous; minor leaves, if present, differing only in size from the majors. Inflorescences 0.1–0.5 cm long, terminal, more or less leaf-opposed or internodal and appearing pseudoaxillary, unbranched or occasionally furcate, with 5–10 flowers, glabrous; peduncle 0.1–0.5 cm long, the flowers in an apical clump; pedicels 0.9–1.1 cm long, < 0.5 mm in diameter at the base and apex, filiform, spreading at anthesis, glabrous, articulated at the base, with a constriction at the apex just below the calyx lobes, this becoming more pronounced in fruit; pedicel scars congested and overlapping at the tip of the very short inflorescence. Buds ovoid, the corolla strongly exserted form the calyx tube before anthesis. Flowers 5-merous, perfect. Calyx tube *ca.* 0.5 mm long, conical, the lobes 0.5–0.75 mm long, *ca.* 0.5 mm wide, deltate, with scarious margins and rounded tips, glabrous. Corolla 0.9–1 cm in diameter, white, stellate, lobed *ca.* 2/3 of the way to the base, the lobes 3–4.5 mm long, 1.5–3 mm wide, spreading or somewhat reflexed at anthesis, the tips and margins minutely papillose. Stamens 2.5–3 mm long; filament tube *ca.* 0.5 mm long, the free portion of the filaments < 0.5 mm long, glabrous; anthers 1.5–2 mm long, *ca.* 1 mm wide, ellipsoid to almost globose, yellow, poricidal at the tips, the pores lengthening to longitudinal slits with age. Ovary glabrous; style *ca.* 4 mm long, glabrous; stigma minutely capitate, the surface papillose. Fruit a globose to somewhat ellipsoidal berry, 0.5–1 cm in diameter, greenish white, occasionally pointed at the apex, the pericarp thin, shiny, brittle when dry; calyx lobes in fruit not markedly enlarging; fruiting pedicels 1–1.3 cm long, 0.5–1 mm in diameter at the base, enlarging gradually to 1.5–2 mm in diameter at the apex, with a slight constriction just below the calyx lobes, not markedly woody, pendant; calyx lobes in fruit not markendly enlarged. Seeds 10–20 per berry, 3–4 mm long, 2–3 mm wide, somewhat flattened-reniform (perhaps immature?), dark to blackish brown, the surfaces minutely pitted, the margins paler and thickened; testal cells pentagonal in outline.

#### Distribution.

south-eastern Brazil in the states of Espirito Santo, Minas Gerais and Rio de Janeiro (Figure 11).

#### Ecology.

*Solanum lacteum* grows in wet Atlantic forests (Mata Atlântica, *Floresta Ombrófila Densa*) in forest understory of well preserved sites, from 600 to 1500 m elevation.

#### Phenology.

No apparent pattern in flowering or fruiting; specimens are often collected with only inflorescences, each plant is very few-flowered.

#### Etymology.

The species epithet was coined by Vellozo (1829) to refer to the whitish colour of the plant – “Color albescens totius plantae nomen triviale dedit” (the white color of the entire plant gives it its trivial name [epithet]). We have not observed entire plants that are white in colour, but suspect Vellozo (1829) was referring to the congested inflorescence that is completely white.

#### Preliminary conservation status (IUCN 2014).

Near Threatened (NT) B1, 2 a, b(ii, iii); EOO 32,466 km^2^ (NT); AOO 28 km^2^ (EN). In spite of its large extent of occurrence, *Solanum lacteum* is only known from six locations and we consider it to be at risk due to the fragmentation and loss of its primary forest habitat. Populations in all three states of occurrence, however, are from within protected areas. It is possible that it is more common than it appears, considering that the flowers are so small and inconspicuous that it is easily overlooked.

#### Notes.

*Solanum lacteum* is characterized by its tiny inflorescences with tightly packed flowers and the difoliate sympodia that are usually not conspicuously geminate. The leaves are narrowly obovate and widest in the distal third. They dry a characteristic blackish brown above and paler brown beneath. The inflorescences often occur internodally and are completely white, including the peduncle and pedicels. The colour of the leaves on herbarium specimens is similar to that of *Solanum caavurana* and *Solanum campaniforme*, but those species always have leaf pubescence on the lower leaf surfaces and more elongate inflorescences. The highly congested inflorescences of *Solanum lacteum* are distinctive and the species is not easily confused with any other growing sympatrically. It is somewhat similar to *Solanum psilophyllum*, which is similarly glabrous; differences between these two species are noted in the discussion of *Solanum psilophyllum*.

*Solanum lacteum* grows in the understory of undisturbed forest and can vary from being a tiny subshrub (see Figure 8B) to a small treelet *ca.* 5 m tall. This variation in height is common in members of the Geminata clade and may have to do with plant age and maturity.

Vellozo’s (1831) illustration (Figure 9) is not particularly clear, but the congested inflorescences and swollen fruiting pedicels with a slight distal constriction are clearly depicted. In addition, *Solanum lacteum* usually has prominent lenticels on the stems; these are also depicted in Vellozo’s plate. We have selected an epitype from Rio de Janeiro State to support this suboptimal plate with material that is fertile and shows the key characters (*Agra et al. 7298*).

We have recognised *Solanum cormanthum* here as a synonym of *Solanum lacteum*; after detailed study we consider the plate of *Solanum cormanthum* (t. 113) to represent flowering material of the same taxon as that shown in fruit in Vellozo’s plate of *Solanum lacteum* (t. 93). *Solanum cormanthum* was used by both Sendtner (1846) and more recently in the *Lista de Especies de Flora do Brasil* (Stehmann et al. 2014) to refer to a different taxon we here recognise as a narrow endemic from Minas Gerais (see *Solanum psilophyllum* below). Both these authors expressed reservations about the correct application of this name. As is the case with the plate of *Solanum lacteum*, the depiction of the plant is not particularly clear, but the small flowers, small anthers and inflorescences that appear axillary (although they are not) are characteristic of *Solanum lacteum*. The locality cited for *Solanum cormanthum* (“silvis maritimis Regii Praedii S. Crucis”; Vellozo 1829: 86) is well within the geographic range and habitat of *Solanum lacteum*, although today it is part of the city of greater Rio de Janeiro.

Sendtner’s (1846) plate of *Solanum glomuliflorum* (f. 11–15) clearly shows the scarious-margined calyx with rounded lobes and very plump anthers characteristic of *Solanum lacteum*. In his protologue Sendtner (1846) cited two collections of *Solanum glomuliflorum*; a flowering specimen of Schott from “Serra d’Estrella” (Serra de Estrela, in Rio de Janeiro State) and a fruiting specimen of Sellow’s from an unspecified locality in Brazil (F neg. 2823; presumably from Berlin]. We select here the Schott specimen at F (accession number 874710; barcode F0073278F) as the lectotype of *Solanum glomuliflorum*, as it bears a label with the locality and collector in J.F. MacBride’s handwriting and presumably comes from Berlin where the original is now destroyed. The collection number 5412 noted on this sheet was not mentioned by Sendtner (1846), but he rarely mentioned collection numbers in his citations.

#### Specimens examined.

**BRAZIL.** Sin. loc., *Herb. Miers 2724* (BM). **Espírito Santo:** Mun. Cariacica, Reserva Biologica Duas Bocas, Alegre, trilha do Pau Oco, 20°17'29"S, 40°31'10"W, 600 m, 4 May 2008 (fr), *A.M. Amorim et al. 7324* (BHCB); Mun. Cariacica, Reserva Biológica de Duas Bocas, localidade de Alegre, trilha do Pau-Oco, 20°17'29"S, 40°31'10"W, 600 m, 20 Jul 2008 (fr), *A.M. Amorim et al. 7563* (BHCB, CEPEC, MBML, RB, UPCB); Mun. Santa Teresa, São Lourenco, Mata do Martinelli, trilha subindo o rio lado direito, 11 Apr 2000 (infl), *V. Demuner et al. 885* (BHCB); Mun. Linhares, Reserva Florestal Linhares, km 0, 23 Jun 1999, *D.A. Folli 3441* (BHCB); Mun. Santa Teresa, Nova Lombardia, terreno de Sr. Furlani, 19°48'14"S, 40°32'17"W, 813 m, 3 Feb 2011 (infl), *L.L. Giacomin et al. 1200* (BHCB); Mun. Santa Teresa, Santo Henrique, terreno Waldecir Frey, 15 Apr 2005 (fr), *L. Kollmann & A.P. Fontana 7642* (BHCB); Mun. Santa Teresa, Nova Lombardia, Reserva Biologica Augusto Ruschi, corrego entre os marcos 130 e 131, 2 Apr 2003 (fl), *R.R. Vervloet & E. Bausen 2110* (BHCB). **Minas Gerais:** Mun. Matão, Estação Biológica de Caratinga, 23 Sep 1984 (fl, fr), *P.M. Andrade & M.A. Lopes 346* (BHCB); Mun. Coronel Pacheco, Estação Experimental de Café Coronel Pacheco, 12 Aug 1941 (fl), *E.P. Heringer et al. 702* (VIC); Mun. Caratinga, Fazenda Montes Claros, Estação Biológica de Caratinga, mata do Rafael, 19°43'53"S, 41°49'02"W, 5 Sep 1998 (fr), *J.A. Lombardi et al. 2334* (BHCB); Mun. Caratinga, Fazenda Montes Claros, 10 Jan 1991 (st), *J.R. Stehmann & C.V. Mendonça s.n.* (BHCB); Mun. Tombos, Fazenda de Cachoeira, 12 Jul 1935 (fl), *Mello Barreto 1577* (BHCB); Mun. Tombos, Mata do Banco, 13 Jul 2007 (fl), *L. Leoni 6947* (BHCB). **Rio de Janeiro:** Mun. Nova Friburgo, RPPN Bacchus, Macaé da Cima, near Nova Friburgo, owned by David and Isabel Miller. Trilha da Antena, 22°22'31"S, 42°29'47"W, 1420 m, 29 Apr 2010 (fl, fr), *M.F. Agra et al. 7296* (JPB, UT); Mun. Rio de Janeiro, Caminho do Macaco, 8 Aug 1878, *A.F.M. Glaziou 9549* (B); Mun. Nova Friburgo, 1883, *A.F.M. Glaziou 14177* (G).

### Solanum psilophyllum

Taxon classificationPlantaeSolanalesSolanaceae

Stehmann & Giacomin sp. nov.

urn:lsid:ipni.org:names:77145589-1

Figure 8C, D, E , 12

#### Diagnosis.

Like *Solanum evonymoides* Sendtn. but differing in smaller flowers, inflorescences that are unbranched or branch only once near the base, pedicels with a constriction at the apex just below the calyx lobes and ovoid-reniform seeds.

#### Type.

Brazil. Minas Gerais: Mun. Mariana, Mina de Fazendão, em mata, próximo à ferrovia, 20°08'43.7"S, 43°24'48.4"W, 875 m, 29 Jul 2008 (fl, fr), *L.L. Giacomin, J.R. Stehmann, S.G. Resende & F. Pena 186* (holotype: BHCB [BHCB019054]; isotypes: BHCB [BHCB019055], BM, NY, RB).

#### Description.

Treelet to 4 m, rhizomatous with underground stems; young stems terete, glabrous; new growth completely glabrous, occasionally minutely papillate; bark of older stems greenish brown, slightly winged from the leaf bases. Sympodial units difoliate, geminate; leaves of a pair differing in size but not usually in shape. Leaves simple, the major leaves 10–15(-25) cm long, 4–13 cm wide, elliptic to narrowly elliptic, occasionally wider in the distal third and narrowly obovate, membranous, glabrous on both surfaces, the abaxial surface often drying paler than the adaxial surface; primary veins 8–11 pairs, drying somewhat lighter than the lamina; base attenuate, somewhat oblique; margins entire; apex acute, the tip somewhat blunt; petiole 1.5–2 cm long, glabrous; minor leaves 6–8 cm long, 2–3 cm wide, differing from the majors only in size and sometimes not present in dried specimens. Inflorescences 0.2–2 cm long, opposite the leaves or appearing to arise from the leaf axils, unbranched, but apparently sometimes with 2 inflorescences from one axil and appearing branched (*Giacomin et al. 186*), with 5–8 flowers, glabrous; peduncle 0.1–2 cm; pedicles 1.2–1.5 cm long, *ca.* 0.5 mm in diameter at the base, *ca.* 1.5 mm in diameter at the swollen apex with a marked constriction just below the calyx lobes, slender and expanding distally, spreading or pendant at anthesis, glabrous, articulated at the base; pedicel scars 0.5 -1 mm apart, more congested in the distal part of the inflorescence. Buds obovoid, the corolla strongly exserted from the calyx tube before anthesis. Flowers 5-merous, perfect. Calyx with the tube 0.5–1 mm long, broadly conical, the lobes 1–1.5 mm long, deltate to triangular, reflexed at anthesis, glabrous. Corolla 1.2–1.4 cm in diameter, white, stellate, lobed 1/2 to 2/3 of the way to the base, the lobes *ca.* 5 mm long, 2.5 mm wide, spread at anthesis, glabrous with the tips minutely papillate. Stamens 3.5–4 mm long; filament tube *ca.* 0.5 mm long, the free portion of the filaments *ca.* 0.5 mm long, glabrous; anthers 2.5–3 mm long, *ca.* 1 mm wide, ellipsoid, yellow, poricidal at the tips, the pores lengthening to slits with age. Ovary glabrous; style 5–6 mm long, glabrous; stigma not expanded, blunt, the surface minutely papillate. Fruit a globose berry, 1–1.3 cm in diameter, green, the pericarp not markedly shiny, thick; fruiting pedicels 1.5–1.7 cm long, *ca.* 1 mm in diameter at the base, 2.5–3 mm and expanded at the apex, woody and pendant; calyx lobes in fruit not markedly expanding, but distinctly differentiated from the enlarged pedicel apex. Seeds not known.

#### Distribution.

In the south-eastern part of the state of Minas Gerais, in islands of forest (*capões*) associated with iron or quartzite formations in the Iron Quadrangle and Serra do Cipó regions, in the southern limit of Espinhaço mountain range (Figure 13).

#### Ecology.

*Solanum psilophyllum* grows in the forest understory on thin soils associated with iron-rich or quartzite formations, at elevations from 800–900 m.

#### Phenology.

Flowering specimens have been collected throughout the year; fruits have only been seen on the type specimen, collected in July. It is probable that this species flowers and fruits sporadically throughout the year.

#### Etymology.

Named for its completely glabrous leaves (from the Greek *psilos* smooth or bare, *phyllos* leaf).

#### Preliminary conservation status (IUCN 2014).

Critically Endangered (CR) B1, 2 a, b(ii, iii, iv); EOO 26 km^2^ (CR); AOO 16 km^2^ (EN). *Solanum psilophyllum* is known from only two localities and its habitat is under severe pressure from mining and frequent forest fires (see Notes). The population from which the type specimen was collected, close to a private railroad, has already been destroyed. Although the area of occupancy would suggest a status of Endangered we consider the extreme threats to these populations coupled with the habitat specifity of members of the Geminata clade (see above) warrant a status of Critically Endangered.One of the known collections might be from a protected area (PARNA Serra do Cipó), although not stated on the specimen label (*Campos & Belisário CFSC-13505*) but appears to be from a roadside, subject to occasional fire.

#### Notes.

*Solanum psilophyllum* is the species previously called *Solanum cormanthum* Vell. in *Lista de Especies de Flora do Brasil* (Stehmann et al. 2014). That name, however, has been of uncertain application since Sendtner (1846) listed a collection from Minas Gerais (“Caxoeira do Campo”) as belonging to *Solanum cormanthum*, but with reservations.

Three sheets of labelled as “Solanum cormanthum Vell.” in Martius’s hand in Brussels belong to this species as do presumed duplicates of this collection in F (F-680206) and G (G00016950) cited by Knapp (2008) as belonging to *Solanum evonymoides* Sendtn., a species now considered to only occur from coastal Bahia to northeastern Minas Gerais (see discussion of *Solanum verticillatum* below). Sendtner (1846) cites a collection in Martius’s herbarium from “Caxoeira do Campo, prov. Minarum, Martio floret: Martius”; this was probably collected by Claussen. One of the three of the sheets in BR (BR00000825373) is from Martius’s herbarium and is labelled “Mart. 1839.” Another sheet is definitely attributed to Claussen and collected in 1835, while the third is attributed (“comm. Schüch fil. 1850”) to Guilherme Schüch, the Baron of Capanema (Minas Gerais, currently the active iron mine of Capanema), who sent plants to Martius.

The Vellozo illustration of *Solanum cormanthum* (tab. 113, Vellozo 1831) has distinctly axillary inflorescences and is said to come from what is now the city of Rio de Janeiro (“Praedii S. Crucis”), an area of very different vegetation and soils than the iron or quartzite rich formations of Minas Gerais. We recognise *Solanum cormanthum* here as a synonym of *Solanum lacteum*, both on morphological and distributional grounds. Members of the Geminata clade are very similar morphologically and Vellozo’s plates are often distinctly suboptimal for secure identification. In view of the restricted distribution and habitat of these plants (see below) we prefer to describe this as new here rather than use *Solanum cormanthum* for these distinct and endangered populations.

*Solanum psilophyllum* has a very narrow distribution restricted to the Iron quadrangle, within areas that are today active mines, and to the Serra do Cipó region, were it was collected more than ten years ago, in forest fragments close to roadsides. The fact that no collections are known from northern areas of the Espinhaço range likely indicates that the distribution is extremely restricted to the region acround Serra do Cipó and the Iron Quadrangle. Efforts to locate new populations of this species are urgent, especially considering that most areas where it might occur are currently owned by mining companies and are subject to an intensive land use.

*Solanum psilophyllum* is morphologically similar to *Solanum verticillatum* (described here below), another completely glabrous species of the Geminata clade occurring in the states of São Paulo and Rio de Janeiro. It can be distinguished from that species by its longer calyx lobes and by the swollen distal portions of the pedicels that are markedly constricted just below the calyx lobes. In addition, the leaf texture of *Solanum psilophyllum* is somewhat fleshy, while leaves of *Solanum verticillatum* are brittle and chartaceous.

*Solanum psilophyllum* is also morphologically similar to *Solanum lacteum* from Atlantic forests in Rio de Janeiro, Espirito Santo and Minas Gerais states. It differs from that species in its larger flowers (>1 cm in diameter), longer inflorescences, elliptic rather than obelliptic leaves that do not dry a blackish brown colour and in the non-lenticellate stem. Like *Solanum lacteum*, *Solanum psilophyllum* is completely glabrous. *Solanum psilophyllum* has an underground stem (Figure 8C), like *Solanum arboreum* Dunal of northern South America and Central America (see Knapp 2002a); this characteristic may be more common in the Geminata clade than currently thought, as it is rare that the underground parts of these small shrubs are collected or even observed.

#### Specimens examined.

**BRAZIL. Minas Gerais:** Mun. Santana do Riacho, Serra do Cipó, Rodovia MG-010, Belo Horizonte a Conceição do Mato Dentro, *ca.* de 1.5 km antes da bifurcação para Morro do Pilar, pequeno capão da mata a direita, próximo a rodovia, 19 Nov 1993 (fl), *M.T.V.A. Campos & A.J.M. Belisário CFSC-13505* (BHCB); sin. loc., 1835 (infl), *P. Claussen s.n.* (BR); sin. loc., 1839 (infl), *P. Claussen s.n.* (F); Caxoeira do Campo, Mar 1839 (infl), *P. Claussen 200* (BR, G); Mun. Santana do Riacho, Serra do Cipó, *ca.* 400 m antes da bifurcação Morro do Pilar-Conceição do Mato Dentro, *ca.* 1.8 km da estrada, 2 Mar 2001 (fl), *M. Groppo et al. 640* (BHCB); Mun. Catas Altas, Mina de Fazendão, próximo à area da cava, 20°07'38"S, 43°24'48"W, 970 m, 27 May 2008 (fl), *S.G. Rezende et al. 2749* (BHCB); sin. loc., 1850 (infl), *G. Schüch s.n.* (BR).

### Solanum verticillatum

Taxon classificationPlantaeSolanalesSolanaceae

S.Knapp & Stehmann sp. nov.

urn:lsid:ipni.org:names:77145590-1

Figures 8F , 14

#### Diagnosis.

Like *Solanum evonymoides* Sendtn. but differing in being a large tree with pseudo-verticillate very shiny chartaceous leaves, smaller, sweet-smelling flowers and orange berries with large seeds.

#### Type.

Brazil. São Paulo: Mun. Santo André, Paranapiacaba, Estação Biológica, 23°46'-23°48'S, 46°21'-46°17'W, 800 m, 30 Jul 1980. *A. Custodio Filho & A.C. Dias 305* (holotype: SP [SP002705]; isotypes: BHCB [BHCB019061], BM [BM001120381]).

#### Description.

Tree to 8 m, the branching appearing somewhat verticillate with branches in congested groups; young stems terete, completely glabrous, usually shiny; new growth completely glabrous and shiny, in live plants sometimes purplish green; bark of older stems pale yellow when dry, in live plants greyish brown. Sympodial units plurifoliate, the leaves clustered along the stems. Leaves simple, 4.5–16 cm long, 2–5 cm wide, elliptic to obelliptic, usually narrowly so, chartaceous and somewhat brittle, both surfaces glabrous and shiny, drying a golden brown; primary veins 6–10 pairs, drying yellowish brown, not looping in a submarginal vein; base acute to acuminate; margins entire, sometimes revolute; apex abruptly acute to attenuate; petiole (0.5-)1–2 cm long, glabrous, drying pale yellowish brown. Inflorescences 2–5 cm long, terminal, appearing axillary but this due to short internodes and congested leaves, branching 1–2 times, with 30–40 flowers, completely glabrous; peduncle 0.5–2.5 cm long; pedicels 1.5–1.7 cm long, *ca.* 0.5 mm in diameter at the base, *ca.* 1 mm in diameter at the apex, filiform, spreading at anthesis, glabrous, articulated at the base; pedicel scars unevenly spaced 1–2 mm apart, usually clustered at the tips of the inflorescence branches. Buds ellipsoid, the corolla completely enclosed in the calyx when young, exserted 2/3 to 3/4 of the way just before anthesis. Flowers 5-merous, all perfect, intensely sweet-smelling (*Custodio Filho 305*). Calyx tube 1–1.5 mm long, conical, the lobes 0.9–1 mm long, *ca.* 1 mm wide, broadly deltate, with scarious margins and a central thickened keel ending in a rounded point, glabrous or the tips with a few papillae. Corolla (1.4-)1.6–1.8 cm in diameter, white, stellate, lobed nearly to the base, the lobes 6–8 mm long, 2.5–3.5(-4) mm wide, spreading at anthesis, densely papillate on the cucullate tips, otherwise completely glabrous. Stamens 4.5–6 mm long; filament tube 1 mm long or less, the free portion of the filaments minute, <0.5 mm long, glabrous; anthers (3-)4–4.5 mm long, 1–1.2 mm wide, obellipsoid with the base narrower than the distal portion, yellow, poricidal at the tips, the pores lengthening to slits with age. Ovary glabrous; style 5–7 mm long, glabrous; stigma minutely capitate, the surface papillose. Fruit a globose berry, 1–1.2 cm in diameter, pale green and white speckled (immature) becoming yellow or orange when ripe, the pericarp shiny and leathery, shattering when pressed and dried; fruiting pedicels 2–2.5 cm long, *ca.* 1 mm in diameter at the base, expanding gradually to *ca.* 2 mm in diameter at the apex, more or less woody, hanging; calyx lobes in fruit not markedly lengthening. Seeds 10–20 per berry, 5–5.5 mm long, 3–4 mm wide, reniform and somewhat flattened, dark brown with paler margins, the surfaces minutely pitted and usually quite thin the embryo easily visible, the testal cells with sinuate margins.

#### Distribution and ecology.

Endemic to south-eastern Brazil, in the states of Minas Gerais, Rio de Janeiro and São Paulo; in the Serra do Mar and Mantiequeira mountain chains (Figure 15).

#### Ecology.

*Solanum verticillatum* grows on the montane coastal forests (Mata Atlântica) as a small tree in forests and secondary growth from 700 to almost 2000 m elevation. Plants can be as large as 10 cm in diameter, and form part of the low canopy of these forests.

#### Phenology.

Most flowering specimens collected in the months of June and July; fruiting in November-January. Sporadic flowering and fruiting apparently occurs throughout the year, but a flowering peak occurs in the austral winter (May-August), which is also the drier season.

#### Etymology.

Named for the pseudo-verticillate nature of the stems, where many branches appear to arise from a set of closely spaced nodes (Figure 8F inset).

#### Preliminary conservation status (IUCN 2014).

Least Concern (LC); EOO 75, 516 km^2^ (LC); AOO 60 km^2^ (EN). Although only described here, *Solanum verticillatum* is known from many localities along the Serra do Mar, many of which are from within protected areas (e.g., Reserva Biológica do Alto da Serra de Paranapiacaba in São Paulo state and Reserva Ecológica de Macaé de Cima, in Nova Friburgo, Rio de Janeiro state). Where it occurs, *Solanum verticillatum* is relatively common.

#### Notes.

*Solanum verticillatum* was considered a montane form of *Solanum evonymoides* by Knapp (2008); field collections in 2013 confirmed the distinctness of this species. *Solanum evonymoides* is known from coastal forests in Bahia and adjacent Espirito Santo, and eastern Minas Gerais and although morphologically similar to *Solanum verticillatum* is distinct in both habitat and in several morphological features. *Solanum verticillatum* differs from *Solanum evonymoides* in its tree habit, branches that appear verticillate due to short internodes (Figure 8F inset), smaller sweet-smelling flowers (< 2 cm in diameter), shiny chartaceous leaves, and orange berries.

*Solanum verticillatum* also resembles *Solanum psilophyllum* (another set of specimens previously recognised as *Solanum evonymoides* by Knapp 2008) in its glabrous shiny leaves. It differs from that species in its more broadly deltate calyx lobes, its distinctly pedunculate inflorescences (versus inflorescences that branch only very near the base in *Solanum psilophyllum*), its berry that is orange or yellow-orange when ripe, and in its flattened rather than ovoid seeds. These two species can be very difficult to distinguish, but the marked constriction just below the calyx lobes at the distal end of the swollen pedicel occurs only in *Solanum psilophyllum*.

This species was commonly collected until approximately the 1980s and populations from the Paranapiacaba reserve are well represented in SP. It is strange that more recent collections do not seem to have been made; this may be due to the tree habit of *Solanum verticillatum* and to its similarity to the more common species *Solanum campaniforme* and *Solanum pseudoquina* A.St.Hil. It can be distinguished from *Solanum campaniforme* by its shiny, completely glabrous leaves (the leaves of *Solanum campaniforme* have tufts of trichomes in the vein axils abaxially) and from *Solanum pseudoquina* by its equal anthers (those of *Solanum pseudoquina* are markedly unequal). It differs from both species in its yellow or orange berries and pseudoverticillate branching. Most specimens of *Solanum verticillatum* at SP were previously identified as *Solanum pseudoquina*.

#### Specimens examined.

**BRAZIL. Minas Gerais:** Mun. Alto Caparaó, Serra do Caparaó, Rancho da Casa Queimada, 2200m, 10 Jul 1941 (fl, fr), *J. de Castro s.n.* (VIC); Mun. Araponga, Parque Estadual da Serra do Brigadeiro, trilha para o Pico do Boné, 26 May 2000 (fr), *A. Salino 5485* (BHCB). **Rio de Janeiro:** Mun. Nova Friburgo, Reserva Macaé de Cima, estrada de terra do Hotel São João para o Sitio dos Miller, 19 Jan 1999 (fr), *L.O. Anderson et al. 99/ 33* (RB); Macaé, Distrito de Frade, trilha para o Pico, 1250 m, 19 Nov 2002 (fr), *M.G. Bovini et al. 2228* (RB); Serra dos Orgãos, 21 Apr 1941 (fl.fr), *A.C. Brade 16776* (RB); Mun. Teresópolis, Teresópolis do Parnaso, excursão a trilha do Rancho Frio, 23K (0704594/7514750), 22°27'16"S, 42°59'19"W, 15 Sep 2010 (fr), *C. Cronemberger et al. 5* (NY, RB); près Theresopolis [no date], *A.F.M. Glaziou 8199* (P); Serra dos Orgãos, près Theresopolis, 1886 (fl), *A.F.M. Glaziou 8856* (G, K); Mun. Nova Iguaçu, Pico do Tinguá, REBIO Tinguá, estrada do Trilha do Rala, Sapé, 22°35'22"S, 42°29'03"W 1600 m (fr), *H.C. de Lima et al. 6006* (RB); Mun. Petrópolis, Araras, 22°23'23"S, 43°13'57"W, 1100 m, 16 Jun 1974 (fl), *G. Martinelli 330* (RB); Mun.Nova Friburgo, Morro da Caledonia, 1400-1600 m, 8 Jun 1977 (fl), *G. Martinelli et al 2440* (K); Mun. Nova Friburgo, Reserva Macaé de Cima, nascente do Rio das Flores, 1000 m, 25 May 1987 (fl, fr), *G. Martinelli et al. 12067* (RB); Mun. Santa Maria Magdalena, Parque Estadual do Desengano, Pedra do Desengano, 21°53'00"S, 41°55'00"W, 1700-1800 m, 21 Dec 1988 (fr), *G. Martinelli et al. 13274* (F, RB); Mun. Santa Maria Magdalena, Parque Estadual do Desengano, Pedra do Desengano, 21°53'00"S, 41°55'00"W, 1800-1850 m, 28 Jun 1989 (fl, fr), *G. Martinelli et al. 13360* (F, RB); Mun. Petrópolis, Serra da Maria Comprida, Distrito de Araras, APA de Petrópolis, João Grande, 22°24'01"S, 43°12'18"W, 1500m, 25 Apr 2006 (fl), *M.A. Moraes & B. Benevenuto RB- 477309* (BHCB); Mun. Nova Friburgo, Reserva Macaé de Cima, trilha em direção ao cume, atrás da casa de Bel e David Muller, 22°00’ S, 42°00’ W, 2 May 2007 (fr), *M.M. Saavedra & M. Bocayuva 381* (BHCB, RB); Mun. Teresópolis, Parque Nacional da Serra dos Orgãos, upper part of the Rancho Frio trail, 22°27'50"S, 43°00'48"W, 1625 m, 8 Mar 2005 (fr), *C. Seele 1004* (RB); Mun. Teresópolis, Parque Nacional da Serra dos Orgãos, vale do Rio Beija-Flor, proximo a trilha da Pedra do Sino, 22°26'53"S, 43°00'20"W, 1265m, 24 Apr 2004 (st), *J.W. Wesenberg 1037* (BHCB); Mun. Teresópolis, Parque Nacional da Serra dos Orgãos, Vale das Orquídeas, 22°27'27"S, 43°01'11"W, 1985 m, 21 Jul 2010 (fr), *J.W. Wesenberg et al. 1046* (RB). **São Paulo:** Mun. São Paulo, desde Parelheiros rumbo a Eng. Marsilac, a 300 m de la Estrada Ponte Seca, 15 Apr 2008, *G.E. Barboza et al. 2025* (CORD); Mun. São Paulo, Marsilac, Parque Estadual Serra do Mar, nucleo Curucutu, caminho para o Mirante, 14 May 1997 (fr), *N.S. Chukr et al. 536* (PMSP); Mun. Itanháem, Parque Estadual Serra do Mar, núcleo de Curucutu, trilha do Rio Camburi, 799m, 15 Mar 2005 (fr), *R. Cielo-Filho et al. 410* (BHCB); Mun. Santo André, Estação Biológica do Alto da Serra de Paranapiacaba, picada 1, 3 Aug 1979 (fl), *A. Custodio Filho et al. 91* (BM, SP); Cunha, Reserva Florestal [44.50-45.50 W, 23.10-23.20S], 1000 m, 11 Jul 1980 (fl), *A. Custodio Filho 265* (BHCB, NY); Mun. São Paulo, Rio Capivari, Distrito de Engo. Marsilac, 23°56'03"S, 46°42'36"W, 800 m, 17 Jun 1992 (fl), *C. Farney et al. 3143* (RB); Mun, São Paulo, Marsilac, Parque Estadual Serra do Mar, nucleo Curucutu, trilha do mirante, 18 Jan 1996 (fr), *G.M.P. Ferreira et al. 35* (BHCB, BM, SP, UEC); Mun. Santo André, Estação Biológica do Alto da Serra de Paranapiacaba [46 21S-46 17S;23 46W-23 28W, DM], 750-790 m, 27 Aug 1980 (fl), *E. Forero et al. 7656* (BM, SP); Mun. Cunha, Parque Estadual Serra do Mar, picada do Rio Bonito, 17 Aug 1994 (fl), *G.A.D.C. Franco & M.L. Kawasaki 1241* (BHCB); Mun. São Paulo, Marsilac, Parque Estadual Serra do Mar, nucleo Curucutu, trilha do Mirante, topo do morro, limite de municipio com Itanhaém, 872m, 13 Apr 1997 (fr), *R.J.F. García & M. Gomes Neto 1161* (PMSP); Mun. Santo André, Paranapiacaba, Parque Municipal das Nascentes de Paranapiacaba, trilha do caminho da Bela Vista, 23°47'21"S, 46°18'11"W, 1056m, 13 Oct 2009 (fr), *L.L. Giacomin et al. 1110* (BHCB, BM); Mun. Santo André, Reserva Biológica do Alto da Serra de Paranapiacaba, 23°46'41"S, 46°18'44"W, 809 m, 19 Nov 2013 (fr), *L.L. Giacomin et al. 2016* (BHCB, BM, UT); Mun. São Paulo, Marsilac, Parque Estadual Serra do Mar, Curucutu, subida para o Mirante, 23°59'28"S, 46°44'36"W, 16 Aug 1995 (fl), *S.A.P. Godoy et al. 755* (BHCB, SP); Mun. Santo André, Alto da Serra (fl), *F.C. Hoehne s.n.* (SP); Mun. Santo André, Alto da Serra, 31 Jul 1918 (fl), *F.C. Hoehne SP-2336* (BM, SP); Mun. Santo André, Alto da Serra, 28 Jan 1919, *F.C. Hoehne 3042* (US); Mun. Santo André, Reserva Biológica do Alto da Serra de Paranapiacaba, área de Campo Grande, 6 Apr 1995 (fr), *M. Kirizawa & E.A. Lopes 2972* (BM,SP); Mun. Santo André, trilha construida pela CESP, estrada da Torre, caminho para o Vale do Quilombo, próximo a Vila de Paranapiacaba, 31 Jan 1996 (fr), *C.Y. Kiyama et al. 103* (SP, UEC); Mun. Santo André, Paranapiacaba, Estação Biológica, 23 May 1946 (fr), *M. Kuhlmann 3420* (BM,SP); Mun. Santo André, Alto da Serra, Estação Biológica, 2 Aug 1928, *D. Lemos s.n.* (BM,SP); Cunha-Res., Est. de Cunha, 11 Jul 1980 (fl), *F.R. Martins et al. 12361* (NY); Mun. Santo André, E.B. Alto da Serra de Paranapiacaba, (via férrea São Paulo-Santos), 28 Oct 1965 (fr), *J. Mattos & C. Moura 12790* (SP); Mun. Santo André, Paranapiacaba, Estação Biológica, via férrea São Paulo-Santos, 30 Sep 1966 (fr), *J. Mattos 13888* (BM, SP);Mun. Santo André, E.B. Alto da Serra de Paranapiacaba, (via férrea São Paulo-Santos), 27 Dec 1966 (fr), *J. Mattos & N. Mattos 14394* (SP); Mun. Santo André, E.B. Alto da Serra de Paranapiacaba, (via férrea São Paulo-Santos), 27 Jul 1967 (fl), *J. Mattos & N. Mattos 14844* (SP); Mun. São Miguel Arcanjo, Parque Estadual Carlos Botelho, estrada de serviço, próximo a Mirante, 2 Sep 2011 (fl, fr), *P.L.R. de Moraes et al. 3327* (BHCB); Mun. Santo André, Alto da Serra, Aug-Sep 1917 (fl), *E. Schwebel 79* (SP); Mun. São Paulo, Parque Estadual Serra do Mar, núcleo Curucutu, trilha do Campo, 23°59'00"S, 46°44'00"W, 800m, 11 Apr 2001 (fr), *F.M. Souza et a.l 63* (BHCB); Mun. Santo André, trilha construida pela CESP, estrada da Torre, caminho para o Vale do Quilombo, próximo a Vila de Paranapiacaba, 31 Jan 1996 (fr), *M. Sugiyama et al. 1403* (BHCB, SP); Mun. Santo André, Paranapiacaba, Estação Biológica, 23°47’ S, 46°19’ W, 750-900 m, 28 Jul 1983 (fl), *C.B. Toledo & A. Custodio Filho 29* (BM, SP).

### Artificial key to the Brazilian species of the Geminata clade

**Note:** Each species’ occurrence in Brazilian states is in square brackets where it keys out. Abbreviations of states follow Table 3.

**Table:** 

1	Mature leaves completely glabrous, with no trichomes > 1 cell long (Note: new growth can have some pubescence in these species)	**2**
–	Mature leaves with at least some trichomes > 1 cell long	**28**
2	Sympodial units plurifoliate, difoliate or unifoliate, not geminate	**3**
–	Sympodial units difoliate and geminate	**14**
3	Sympodial units unifoliate; new growth with minute branched trichomes [BA; ES; MG]	***Solanum bahianum***
–	Sympodial units with more than one leaf; new growth glabrous or with arachnoid (tangled like spider’s webs) or scurfy pubescence	**4**
4	Stems winged [PR; RS; SC]	***Solanum alatirameum***
–	Stems not strongly winged	**5**
5	Inflorescence many times branched	**6**
–	Inflorescence simple or at most once-branched (often near the base)	**11**
6	Leaves with conspicuous domatia like small pits in the vein axils abaxially [PR; RS; SC; SP]	***Solanum pabstii***
–	Leaves without domatia abaxially	**7**
7	Corolla < 1 cm in diameter; leaf bases acute or cuneate; plants often drying black or dark brown [BA]	***Solanum cordioides***
–	Corolla > 1 cm in diameter; leaf bases attenuate; plants not drying black or dark brown	**8**
8	Inflorescences stout, the pedicel scars closely spaced and usually overlapping; leaves large and repand with parallel venation; new growth with brown scurfy pubescence [AC; AM]	***Solanum robustifrons***
–	Inflorescences not stout, the pedicel scars not overlapping; leaves not repand with parallel venation; new growth glabrous or with minute golden pubescence, not scurfy and reddish brown when dry	**9**
9	New growth and inflorescence axes with minute golden pubescence; leaves matte, sessile or very short petiolate; buds completely enclosed in the calyx when young [AC; AM]	***Solanum sessile***
–	New growth and inflorescence axes glabrous and shiny; leaves shiny, petiolate; buds not completely enclosed in calyx	**10**
10	Leaves chartaceous, apparently whorled, wider in the distal third; flowers sweet-smelling; mature fruit orange or yellow; montane areas [MG; RJ; SP]	***Solanum verticillatum***
–	Leaves membraneous to fleshy, not whorled, widest in the middle; flowers not sweet-smelling; mature fruit green; coastal [BA; ES; MG; RJ]	***Solanum evonymoides***
11	New growth with arachnoid or scurfy pubescence	**12**
–	New growth completely glabrous	**13**
12	New growth with matted arachnoid pubescence; sympodial units plurifoliate; inflorescences simple [PR; SC]	***Solanum canoasense***
–	New growth with scurfy papillate pubescence; sympodial units difoliate; inflorescences simple or furcate AC; AM]	***Solanum robustifrons***
13	Flowers > 1 cm in diameter; inflorescence > 1 cm long; leaves elliptic [MG]	***Solanum psilophyllum***
–	Flowers < 1 cm in diameter; inflorescence < 1 cm long; leaves obelliptic [ES; MG; RJ]	***Solanum lacteum***
14	Leaves of a geminate pair not differing markedly in shape (but can differ in size)	**15**
–	Leaves of a geminate pair differing markedly in shape (usually also in size)	**20**
15	New growth finely golden pubescent; plants of the Amazon [AC; AM; PA; RR]	***Solanum oppositifolium***
–	New growth glabrous; plants of SE Brazil	**16**
16	Stems strongly winged inflorescence branched [PR; RS; SC]	***Solanum alatirameum***
–	Stems terete, not winged; inflorescence unbranched or at most furcate	**17**
17	Inflorescences elongate (> 2 cm long), the pedicel scars not overlapping; pedicels strongly winged [BA; ES; MG; RJ]	***Solanum warmingii***
–	Inflorescence minute (< 0.5 cm long), the pedicel scars overlapping; pedicels terete	**18**
18	Stems strongly winged [BA; ES; RJ]	***Solanum restingae***
–	Stems terete	**19**
19	Calyx lobes narrowly triangular, 1–1.5 mm long; corolla with the lobes reflexed; stem not lenticellate; leaves not drying black or dark brown [BA; MG]	***Solanum amorimii***
–	Calyx lobes deltate, < 1 mm long; corolla with the lobes spreading; stem strongly lenticellate; leaves drying black or dark brown [ES; MG; RJ]	***Solanum lacteum***
20	Fruits red or orange; fruiting pedicels erect	**21**
–	Fruits green or yellowish green; fruiting pedicels deflexed	**22**
21	Flowers > 1 cm in diameter, the corolla lobes spreading; fruit dark orange or red [cultivated]	***Solanum pseudocapsicum***
–	Flowers < 1 cm in diameter, the corolla lobes strongly reflexed; fruit pale orange [cultivated]	***Solanum diphyllum***
22	Stems winged	**23**
–	Stems terete	**24**
23	Bark of older stems white and peeling; internodes crowded; inflorescences filiform and pedicel scars spaced; flowers < 1 cm in diameter; plants of river courses [BA; ES; MG; PR; RJ; SC; SP]	***Solanum stipulatum***
–	Bark of older stems not markedly peeling; internodes not crowded; inflorescences very short and thick and pedicel scars congested; flowers > 1 cm in diameter; plants of forest understory [BA; ES; RJ]	***Solanum restingae***
24	Buds ellipsoid or turbinate; corolla > 1.5 cm in diameter, the lobes cucullate, spreading; minor leaves usually heart-shaped	**25**
–	Buds globose; corolla < 1 cm in diameter, the lobes not cucullate, reflexed; minor leaves not usually heart-shaped	**26**
25	Buds turbinate; calyx lobes deltate or broadly deltate; fruiting pedicels swollen at distal end just below the calyx; new growth finely pubescent with whitish trichomes [AC; AM; GO; MA; MG; MT; PA; RO; RR]	***Solanum leucocarpon***
–	Buds ellipsoid; calyx lobes long-acuminate; fruiting pedicels gradually tapering to apex; new growth glabrous [AC; AM]	***Solanum anisophyllum***
26	Inflorescence stout; pedicel scars closely spaced; fruiting pedicels erect [RR]	***Solanum arboreum***
–	Inflorescence filiform; pedicel scars evenly but not tightly spaces; fruiting pedicels deflexed	**27**
27	Minor leaves very small and appearing stipulate; new growth and calyx lobes with fine golden pubescence (this occasionally extending to the midrib); plants of the Amazon [AM]	***Solanum leptopodum***
–	Minor leaves not stipulate; new growth and calyx lobes glabrous; plants of Mata Atlântica [PE]	***Solanum* sp. 1**
28	Trichomes variously branched	**29**
–	Trichomes simple or at most a few furcate	**41**
29	Upper leaf surfaces glabrous and shiny; if trichomes present then the upper surface very sparsely pubescent	**30**
–	Upper leaf surfaces not markedly shiny; variously pubescent	**33**
30	Trichomes lax and dendritic	**31**
–	Trichomes with more densely congested branches (or echinoid)	**32**
31	Leaves sessile or the base strongly attenuate; trichomes sparse on lower leaf surface [PR; SC; SP]	***Solanum pseudodaphnopsis***
–	Leaves petiolate; trichomes dense on lower leaf surface, obscuring the lamina [RJ; RS]	***Solanum arenarium***
32	Trichomes in axillary tufts; stems glabrous or only sparsely pubescent with mostly uniseriate trichomes on new growth; plants of the Amazon [AC; AM]	***Solanum nudum***
–	Trichomes distributed over entire abaxial lamina; stems densely to moderately pubescent with dendritic trichomes; plants of south-eastern Brazil	**33**
33	Inflorescence simple; sympodial units difoliate, geminate or not geminate [BA; ES; MG; RJ]	***Solanum kleinii***
–	Inflorescence several to many times branched sympodial units plurifoliate [PR; RS; SC]	***Solanum compressum***
34	Pedicels distinctly swollen at the distal end; flowers fleshy, the corolla lobes spreading [AC; AM; GO; MA; MG; MT; PA; RO; RR]	***Solanum leucocarpon***
–	Pedicels tapering to the distal end; flowers not markedly fleshy, corolla lobes spreading or reflexed	**35**
35	Mature fruit green or yellowish green; flowering and fruiting pedicels nodding or spreading	**36**
–	Mature fruit red or orange; fruiting pedicels erect; flowering pedicels nodding	**38**
36	Inflorescence many times branched; sympodial units plurifoliate [PR; RS; SC]	***Solanum compressum***
–	Inflorescence simple; sympodial units defoliate	**37**
37	Leaf trichomes whitish in colour; sympodial units difoliate, not geminate; flowers fleshy; fruit glabrous [MG; PR; RS; SC]	***Solanum cassioides***
–	Leaf trichomes beige or brownish in colour; sympodial units difoliate, geminate and anisophyllous; flowers membranous; fruit densely pubescent [MG; PR; RJ; SP]	***Solanum gnaphalocarpon***
38	Sympodial units di-or trifoliate; pubescence a mixture of simple and dendritic trichomes [PR; RS; SC; SP]	***Solanum delicatulum***
–	Sympodial units defoliate; pubescence of only dendritic trichomes	**39**
39	Leaves narrowly linear [SP]	***Solanum spissifolium***
–	Leaves elliptic	**40**
40	Trichomes reddish brown, 1–2 mm long, evenly distributed on both leaf surfaces [PR; SC; SP]	***Solanum kleinii***
–	Trichomes whitish cream, 0.25–0.5 mm long, denser abaxially [plants from natural habitats, not cultivated; DF; ES; GO; MG; MS; MT; PR; RJ; RS; SC; SP]	***Solanum pseudocapsicum***
41	Leaf trichomes evenly distributed on both surfaces, always extending to the lamina abaxially	**42**
–	Leaf trichomes confined to the abaxial surfaces; often in tufts in the vein axils (if pubescence on upper surface then this very sparse and only along the midrib)	**44**
42	Trichomes < 1 mm long, 1–2-celled, from broad multicellular bases, hooked; leaves scabrous [PR; RS; SC; SP]	***Solanum trachytrichium***
–	Trichomes > 1 mm long, if less than 1 mm long then multi-celled, not hooked; leaves not scabrous	**43**
43	Leaves only sparsely pubescent above; trichomes white, minute; pedicel with an expanded distal end; flowers fleshy, the corolla lobes spreading [MS; MT; RO]	***Solanum corumbense***
–	Leaves evenly pubescent on both surfaces; trichomes translucent, to 2 mm long; pedicel filiform; flowers membraneous, the corolla lobes reflexed [PR; SC; SP]	***Solanum apiahyense***
44	Pubescence evenly distributed over entire lower leaf surface	**45**
–	Pubescence confined to tufts in leaf vein axils or along the midrib	**46**
45	Anthers unequal; pores never lengthening to slits [PR; RS; SC]	***Solanum reitzii***
–	Anthers of equal size; pores lengthening to slits with age [MG; RJ; SP]	***Solanum intermedium***
46	Flowers > 1.5 cm in diameter, somewhat fleshy; corolla lobes spreading	**47**
–	Flowers < 1.5 cm in diameter, not markedly fleshy; corolla lobes reflexed or spreading	**50**
47	Calyx lobes expanded and petaloid; pedicels tapering evenly from base to tip	**48**
–	Calyx lobes variously deltate or triangular, not petaloid or markedly expanded; pedicels with a swollen distal end	**49**
48	Pedicels strongly winged, green [BA; ES; MG; RJ]	***Solanum warmingii***
–	Pedicels terete, white [AL; BA; CE; ES; MA; MG; MS; MT; PB; PE; PI; PR; RJ; RN; SC; SE; SP]	***Solanum caavurana***
49	Leaf base abruptly attenuate [MS; MT; RO]	***Solanum corumbense***
–	Leaf base acute [AC; AM; GO; MA; MG; MT; PA; RO; RR]	***Solanum leucocarpon***
50	Bark of older stems (not very new growth) pale white or yellowish green (especially when dry)	**51**
–	Bark of older stems (not very new growth) brown or grey, not yellowish green	**52**
51	Stems with long multicellular trichomes; flowers with equal anthers and filaments; anther pores opening to slits [PR]	***Solanum gertii***
–	Stems glabrous; flowers with unequal anthers and filaments; anther pores round [BA; ES; MG; PR; RJ; RS; SC; SP]	***Solanum pseudoquina***
52	Inflorescences elongate and filiform, with widely spaced pedicel scars	**53**
–	Inflorescences not elongate or filiform, the pedicel scars closely spaced or overlapping	**54**
53	Leaf margins undulate (ruffled); flowers < 1 cm in diameter, the corolla lobes not markedly cucullate [ES]	***Solanum filirhachis***
–	Leaf margins plane; flowers > 1 cm in diameter, the corolla lobes cucullate [AM; BA; CE; DF; ES; MA; MG; PA; PB; PE; PR; RJ; RR; RS; SC; SP]	***Solanum campaniforme***
54	Flowers congested at apex of inflorescence; pedicel scars overlapping; calyx lobes long triangular [MG; MT; PR]	***Solanum symmetricum***
–	Flowers spaced along the inflorescence axis; pedicel scars closely spaced, but not markedly overlapping; calyx lobes deltate or spathulate	**55**
55	Stems winged; calyx lobes spathulate; plants of south-eastern Brazil [BA]	***Solanum santosii***
–	Stems terete; calyx lobes deltate; plants of the Amazon [AC; AM]	***Solanum nudum***

## Supplementary Material

XML Treatment for Solanum amorimii

XML Treatment for Solanum apiahyense

XML Treatment for Solanum filirhachis

XML Treatment for Solanum lacteum

XML Treatment for Solanum psilophyllum

XML Treatment for Solanum verticillatum
